# Cell unit-inspired natural nano-based biomaterials as versatile building blocks for bone/cartilage regeneration

**DOI:** 10.1186/s12951-023-02003-0

**Published:** 2023-08-24

**Authors:** Fuxiao Wang, Zhengrong Gu, Zhifeng Yin, Wencai Zhang, Long Bai, Jiacan Su

**Affiliations:** 1 Institute of Translational Medicine, Shanghai University, Shanghai, 200444 China; 2 Department of Orthopedics, Shanghai Baoshan Luodian Hospital, Baoshan District, Shanghai, China; 3 Department of Orthopedics, The Third Affiliated Hospital of Guangzhou University of Traditional Chinese Medicine (TCM), Guangzhou, China; 4 Department of Orthopedics, Shanghai Zhongye Hospital, Shanghai, China

**Keywords:** Cell unit, Natural nano-based biomaterials, Building blocks, Bone regeneration, Cartilage regeneration

## Abstract

The regeneration of weight-bearing bone defects and critical-sized cartilage defects remains a significant challenge. A wide range of nano-biomaterials are available for the treatment of bone/cartilage defects. However, their poor compatibility and biodegradability pose challenges to the practical applications of these nano-based biomaterials. Natural biomaterials inspired by the cell units (e.g., nucleic acids and proteins), have gained increasing attention in recent decades due to their versatile functionality, compatibility, biodegradability, and great potential for modification, combination, and hybridization. In the field of bone/cartilage regeneration, natural nano-based biomaterials have presented an unparalleled role in providing optimal cues and microenvironments for cell growth and differentiation. In this review, we systematically summarize the versatile building blocks inspired by the cell unit used as natural nano-based biomaterials in bone/cartilage regeneration, including nucleic acids, proteins, carbohydrates, lipids, and membranes. In addition, the opportunities and challenges of natural nano-based biomaterials for the future use of bone/cartilage regeneration are discussed.

## Introduction

Bone and cartilage defects are prevalent clinical conditions that significantly impair functionality and limit quality of life. It is estimated that approximately 15 million fractures and 500,000 knee operations occur annually, highlighting the need for effective treatment options [1]. Repairing and regenerating these defects presents a significant challenge for clinicians [2]. While traditional treatments such as autografts and allografts have brought new hope for the regenerative treatment of bone and cartilage defects, most of these strategies have limitations and complications despite avoiding immunogenicity [3]. Autografts, for example, are costly, can cause pain and infections, and face serious problems with infectious transmission from the donor [4].

Over the past two decades, there has been significant progress in the development of bone and cartilage tissue engineering with the application of implantable synthetic nano-based biomaterials [5]. However, these nano-based biomaterials may lead to suboptimal or detrimental outcomes due to their material characteristics. For instance, metal-based materials such as titanium and magnesium have been identified as nano-based biomaterials for bone repair but face challenges with insufficient bioactivity, implant loosening, and fast degradation [6, 7]. Bioceramic materials such as calcium orthophosphate compounds and beta tricalcium phosphate have been proposed as synthetic bone graft substitutes, but sintered bioceramics are non-biodegradable and hardly bioactive, making it challenging to apply widely in clinical practice. Additionally, there are no load-bearing applications for calcium phosphate ceramics due to poor mechanical properties [8]. Likewise, piezoelectric materials such as zinc oxide and boron nitride require further investigation to evaluate their cytotoxicity and ensure their safety for use in biomedical applications [9, 10]. Therefore, discovering a biomaterial with a high degree of histocompatibility and minimal side effects is highly desired.

In recent decades, natural nano-based biomaterials have been widely recognized as ideal candidates due to their functional diversity, compatibility, biodegradability, and potential for modification, compounding, and hybridization [11]. Natural nano-based biomaterials are materials found in nature, such as proteins, carbohydrates, lipids, nucleic acids, and other molecules, that are also the building bio-blocks of the cell. Biomacromolecules, such as nucleic acids (DNA and RNA), proteins (collagen and silk fibroin), carbohydrates (cellulose, dextran, agarose, starch, alginate, hyaluronic acid, chondroitin sulfate, heparin, gellan gum, chitin and chitosan) and lipids (liposome), which are basic elements of cells. And cell membrane is based on the organization of lipids and protein/glycoprotein complexes. Natural nano-based biomaterials can be used in both bone and cartilage applications, but they must be customized to meet the specific needs of each tissue. Nano-based biomaterials for bone regeneration must be able to withstand compressive forces, promote bone formation, and integrate with surrounding tissue, while nano-based biomaterials for cartilage regeneration must withstand tensile and compressive forces, promote cartilage formation, and mimic the mechanical properties of native cartilage. Hence, to choose proper nano-based biomaterials for bone/cartilage regeneration, many factors should be considered.

These natural nano-based biomaterials would be promising in bone/cartilage regeneration as they not only possess the biocompatibility and favorable mechanical properties, but also increase the possibilities of cell adhesion/proliferation/differentiation, cell/tissue targeting, anti-inflammatory action, and so on. For example, collagen fibrillation and a sacrificing material (pluronic F-127) constitute 3D collagen scaffolds were nontoxic and can effectively promote osteogenic differentiation [12]. Hydroxyapatite (HA) concatenated with vascular endothelial growth factor (VEGF) specifically targeted DNA aptamer could promote bone regeneration [13].

**Fig. 1:**
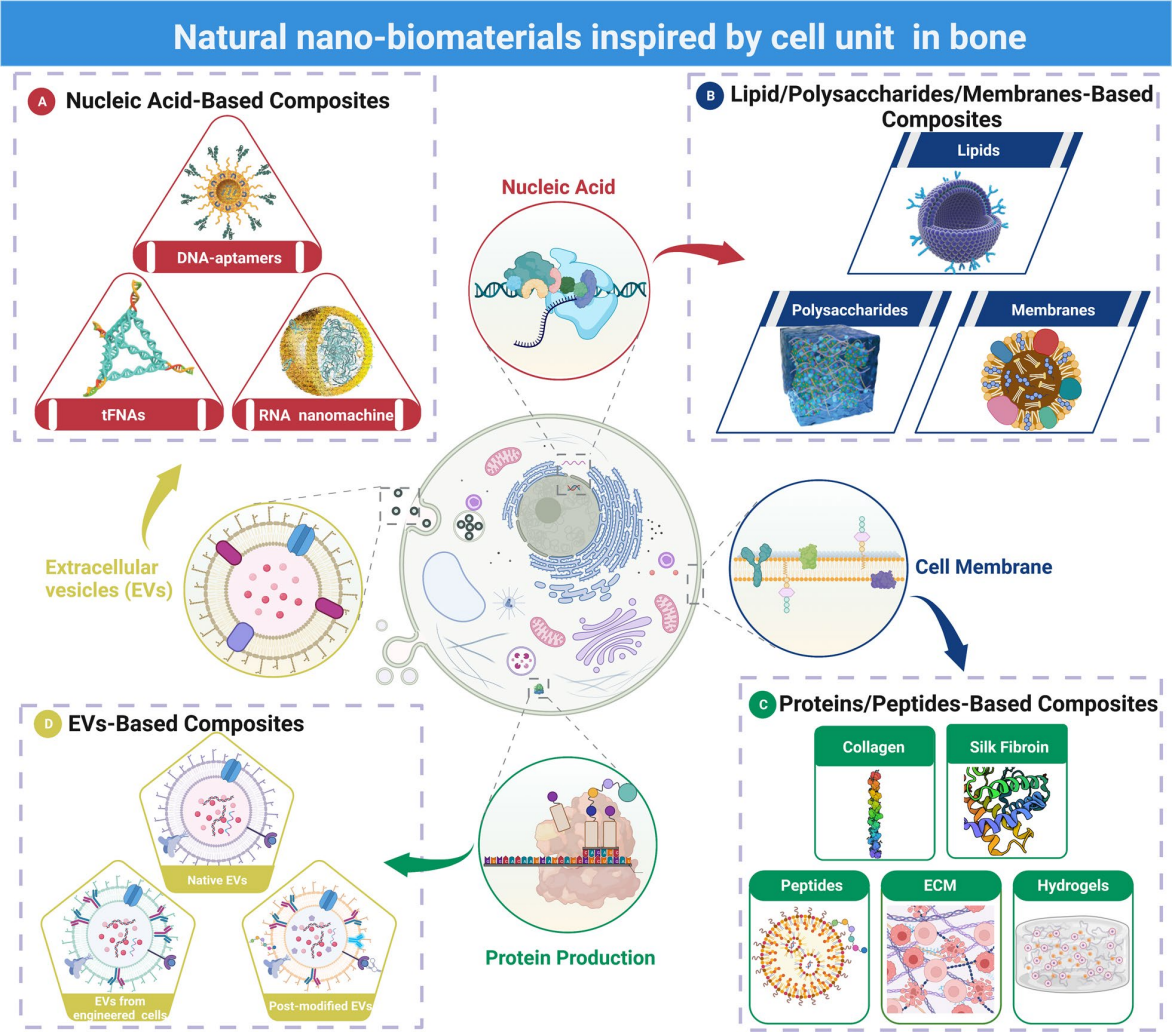
Schematic representation of natural nano-based biomaterials inspired by cell unit in bone. **A** Nucleic acids-based composites, such as DNA aptamers, tFNAs (tetrahedral framework nucleic acids) and RNA nanomachine. **B** Cell membrane is based on the organization of lipids and protein/glycoprotein complexes. Each of the biomacromolecules in the complex, as well as the cell membrane itself, works well in bone/cartilage regeneration. **C** Proteins, such as collagen and silk fibroin, and peptides-based composites have been effectively used in bone/cartilage regeneration. **D** Extracellular vesicles (EVs) are of cellular origin, they can be grouped into native EVs, EVs from engineered cells and post modified EVs. Created with BioRender.com

Herein, we systematically summarize the versatile building blocks inspired by the cell unit used as natural nano-based biomaterials in bone/cartilage regeneration, including nucleic acids, proteins, carbohydrates, lipids, and membranes. We also categorize these natural nano-based biomaterials according to some of their properties and discuss the potential applications of these natural nano-based biomaterials in the treatment of bone diseases and provide insight into their future development (Fig. 1A–D).

## Cell unit-inspired building blocks for bone/cartilage regeneration

### Nucleic acids-inspired building blocks

**Fig. 2:**
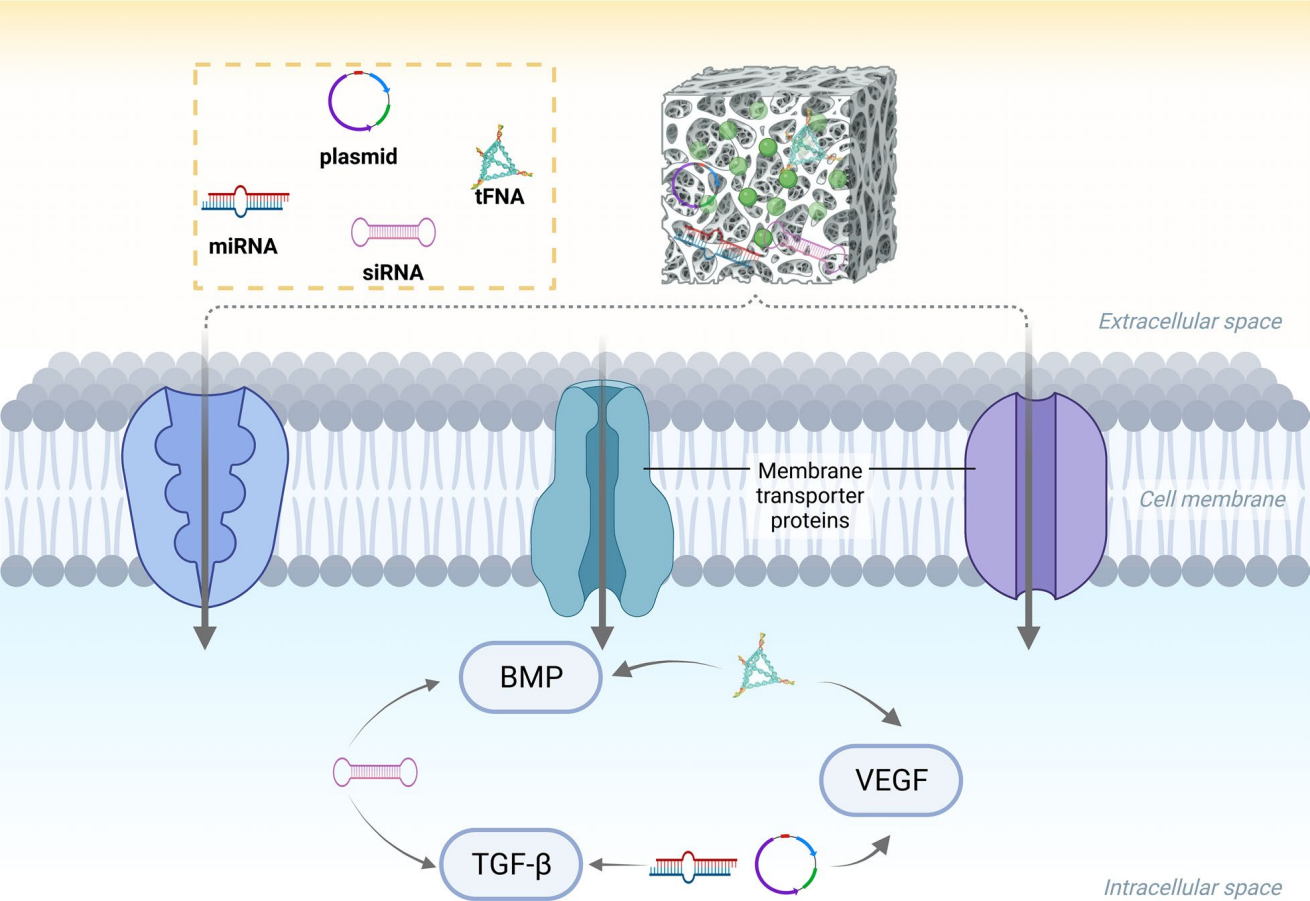
Schematic representation of target of nucleic acid following delivery to cells

Nucleic acids are fundamental biomolecular compounds composed of nucleotide monomers, and are essential building blocks of life. There are two main types of nucleic acids, namely deoxyribonucleic acid (DNA) and ribonucleic acid (RNA). DNA is a crucial macromolecule that carries genetic information, which is responsible for the synthesis of RNA and proteins. For bone/cartilage regeneration, nucleic acid delivery systems, including exogenously engineered genes or nucleic acids (DNA or RNA) and nucleic acid analogs (peptide nucleic acids or locked nucleic acids), offer unique advantages. They can be designed to deliver therapeutic genes or small interfering RNA (siRNA) molecules to bone cells, enabling the modulation of specific cellular processes [14]. These systems are often combined with biomaterial scaffolds or carriers to provide structural support, protect the nucleic acids from degradation, and facilitate their localized delivery to the target site (Fig. 2).

Apart from being used as therapeutic drugs, nucleic acids can also serve as natural biomaterials for bone and cartilage regeneration. For example, DNA's sequence-specific hybridization has made it widely applicable in biosensing and biomedical engineering [15]. RNA, on the other hand, serves as a messenger for protein production, due to the different order of nucleobases on its backbone. The investigation of RNA aptamers and RNA-biomineral nanomachines continues to gain momentum in bone/cartilage research [16, 17].

#### DNA

**Fig. 3:**
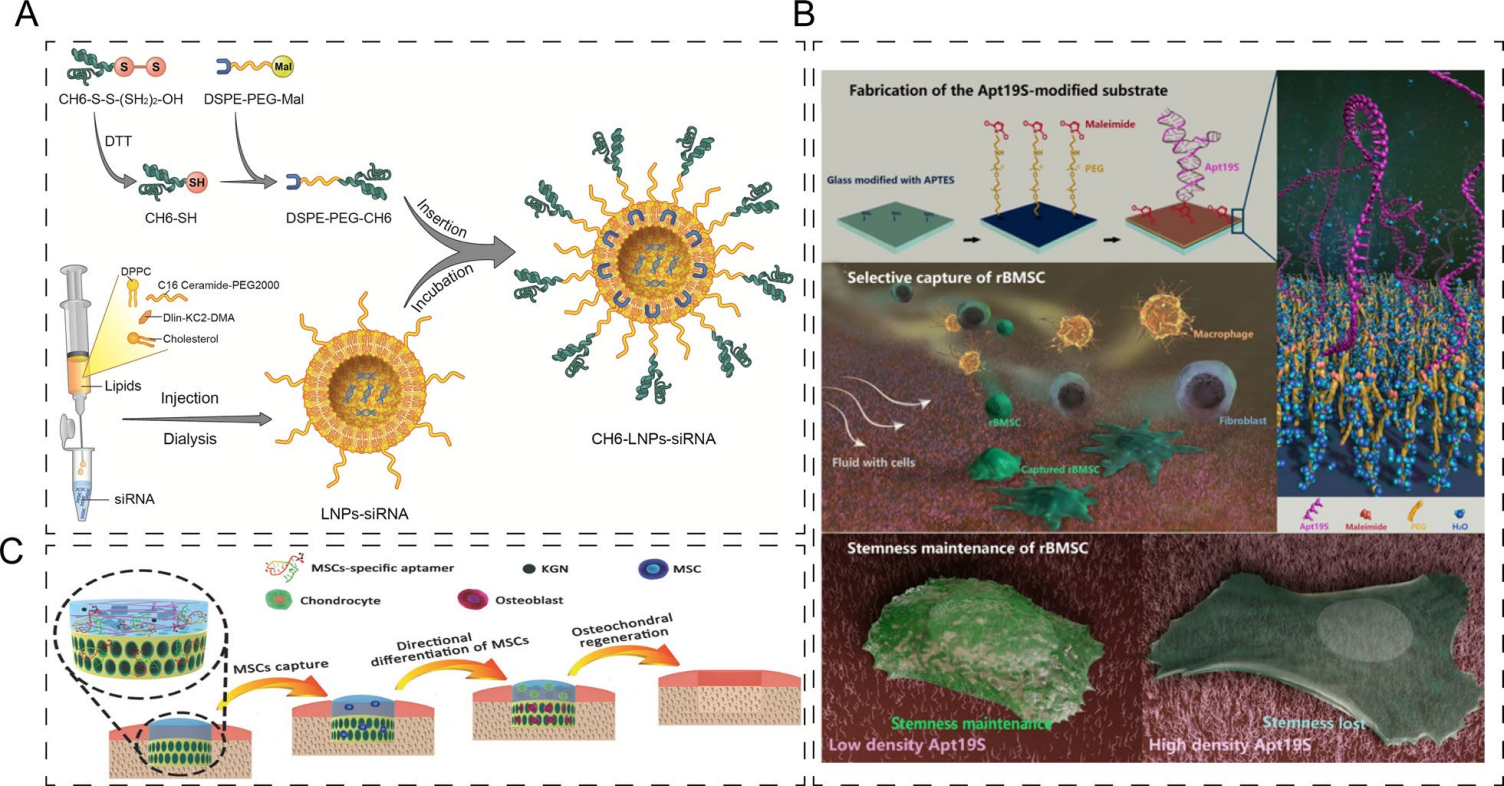
Application of DNA aptamers-based cells targeting strategies in bone/cartilage regeneration. **A** Osteoblast-specific aptamers CH6 functionalized lipid nanoparticles for delivering osteogenic siRNAs. Reprinted with permission [21]. **B** Schematic illustration of the process of Apt19 modification and cell adhesion assay. Reprinted with permission [23]. **C** Apt19 immobilized bilayer scaffold for MSCs capture. Reprinted with permission [24]

Aptamers are unique ligands with specific sequences obtained through in vitro selection or systematic evolution of ligands by exponential amplification (SELEX), linking genotype and phenotype. DNA aptamers are single-stranded oligonucleotide structures that can selectively bind to targeted molecules and materials due to their high selectivity and strong stability [18, 19]. As such, DNA aptamers are frequently used to deliver therapeutic drugs to bone defect sites by targeting specific types of cells [20]. For example, Ge Zhang et al. [21] used cell-SELEX to develop osteoblast-specific aptamers CH6, which were functionalized on lipid nanoparticles for osteogenic siRNAs, resulting in enhanced gene silencing and bone anabolic effects. The delivery systems based on osteoblast-specific aptamers CH6 were able to target cells at the cellular level (Fig. 3A). Apt19 is another DNA aptamer that specifically labels multipotent stem cells and can be used as an affinity reagent for stem cell enrichment [22]. By modifying the Apt19S on a polyethylene glycol (PEG) layer, the cell adhesion assay showed an increase in the adhesion ratio of rat bone-marrow-derived MSCs (rBMSCs) and selective adsorption of rBMSCs in vitro (Fig. 3B) [23]. Quan Yuan et al. [24] immobilized Apt19 on a bilayer scaffold to capture mesenchymal stem cells (MSCs) for the differentiation of chondrocytes. The Apt19-functionalized scaffold recruited endogenous MSCs to osteochondral defect sites and significantly repaired the joints (Fig. 3C). Apart from its application in cartilage regeneration, Apt19 can also be used to induce bone formation in osteoporosis by binding with the alkaline phosphatase on MSC surface. The presence of p-OGP (phosphorylated osteopontin) and hydroxyapatite at the implant interface significantly enhances the osteogenic differentiation potential of MSCs, promoting effective bone regeneration [25].

DNA aptamers have been explored as potential therapeutics for bone and cartilage regeneration due to their ability to bind to growth factors, cytokines, and other signaling molecules involved in bone and cartilage formation and regeneration. However, there are several challenges associated with the application of DNA aptamers in bone/cartilage regeneration. For example, DNA aptamers are susceptible to degradation by nucleases, which can limit their effectiveness and bioavailability. To overcome this, modifications to the aptamer sequence may be necessary to increase stability, such as chemical modifications or the incorporation of protective groups.

**Fig. 4:**
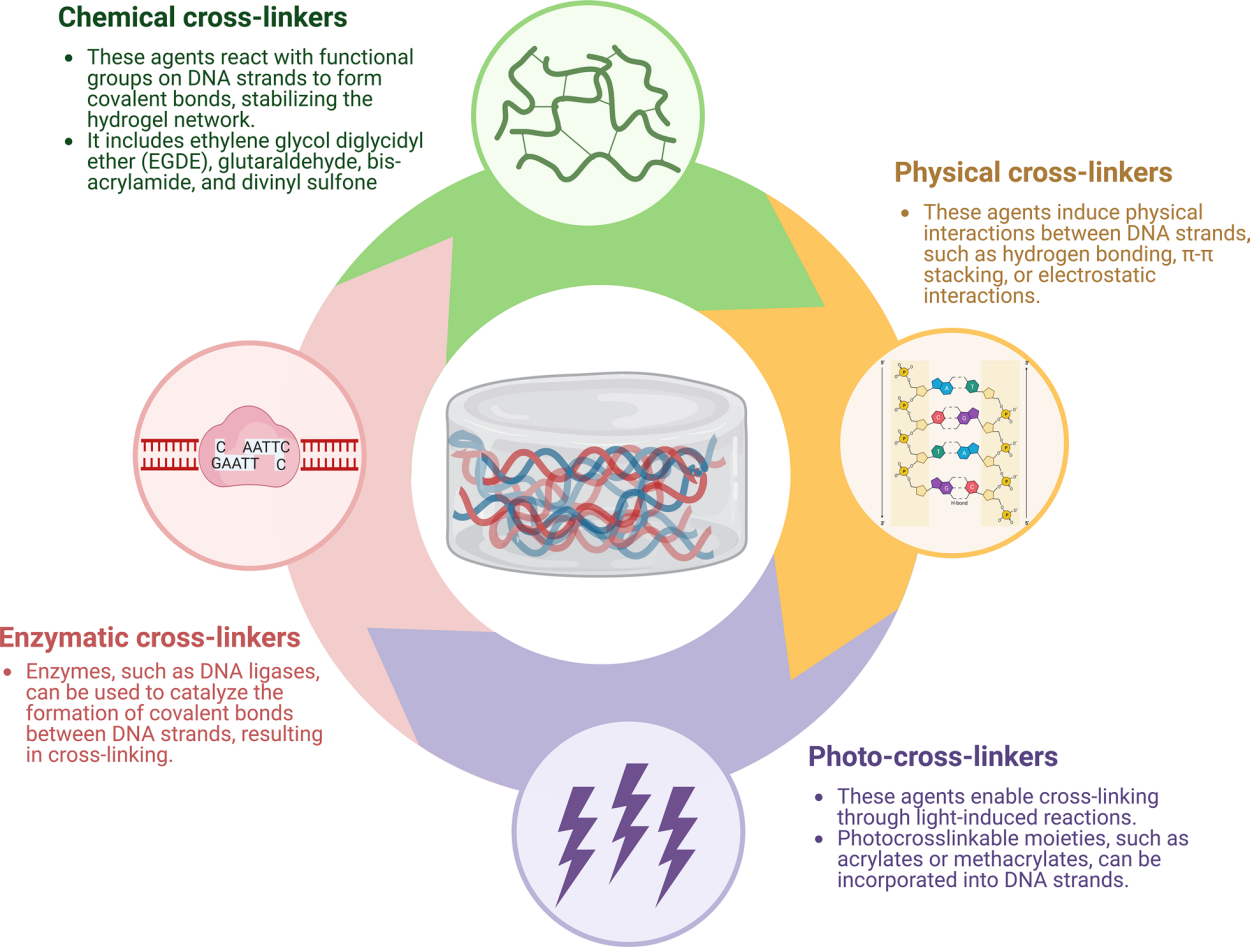
The cross-linking agents in DNA hydrogel

DNA hydrogels have emerged as promising materials for bone and cartilage regeneration, where DNA acts as either crosslinkers or responsive units. Two types of DNA hydrogels have been explored so far: pure DNA hydrogels and hybrid DNA hydrogels [26]. Pure DNA hydrogels, owing to their biodegradability and biocompatibility, have demonstrated exceptional functions in biomedical applications. For instance, to treat osteoarthritis (OA), DNA hydrogels have been employed to transport BMSCs to the defect sites, thereby providing a 3D microenvironment for cell proliferation and reducing friction, which mitigated the consumption of BMSCs due to the shear forces between the contact cartilage faces [27]. In another approach, Wang et al. connected VEGF-decorated black phosphorus nanosheets (BPNSs) with DNA hydrogels to enhance their mechanical strength [28]. After incorporating them into 3D-printed poly-ε-caprolactone (PCL) scaffolds, the DNA hydrogel composite materials accelerated bone tissue regeneration by ensuring high loading efficiency and sustained release profile of VEGF. The DNA backbone has also been utilized to develop sustained drug release hydrogels, as demonstrated by Paul et al. [29] Their study showed that DNA-based nanocomposite hydrogels facilitated the sustained release of dexamethasone and promoted osteogenic potential in vivo. DNA hydrogels are three-dimensional polymeric networks composed of DNA and a cross-linking agent (Fig. 4) [30–33]. The mechanical properties of DNA hydrogels can be difficult to control and optimize for specific applications. Hydrogels that are too soft may not provide enough support for tissue regeneration, while hydrogels that are too stiff may impede cellular infiltration and tissue integration. Therefore, how to control the variables so that DNA hydrogels have the most suitable mechanical parameters is one of the key considerations.

**Fig. 5:**
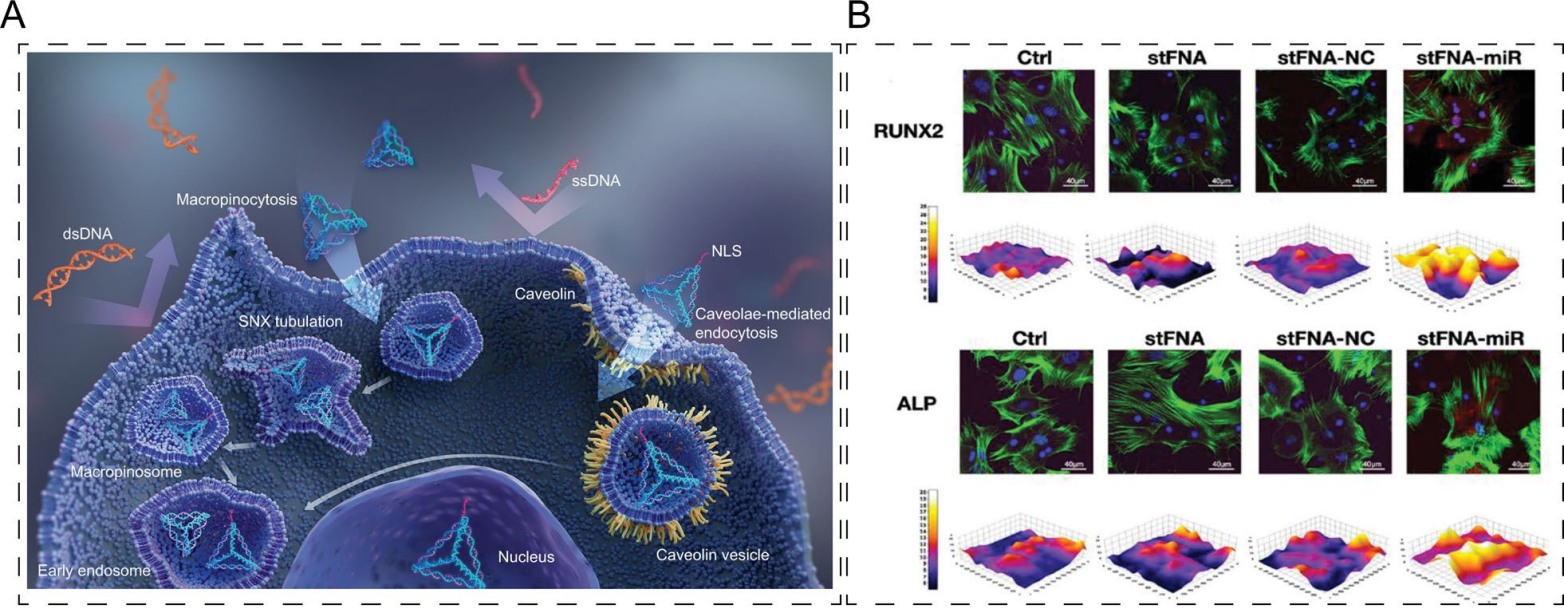
Application of tFNAs-based composites in bone/cartilage regeneration. **A** Schematic illustration of tFNAs entered the cell and NLS functioned tFNA entered the nucleus. Reprinted with permission [36]. **B** miR-2861 loaded tFNAs promoted the expression of Runx2 and ALP identified by immunofluorescence. Reprinted with permission [37]

Tetrahedral framework nucleic acids (tFNAs), also known as tetrahedral DNA nanostructures, possess unique structural stability, tissue permeability, size-dependent tissue penetration, and low immunogenicity [34, 35]. Due to their high level of negative charges, DNA cannot penetrate cell membranes autonomously [36]. However, tFNAs are able to enter cells, and nuclear localization signal (NLS)-functionalized tFNAs have been demonstrated to enter the nucleus (Fig. 5A). tFNAs are particularly effective in transporting cargo, such as RNA or DNA molecules, into cells. For example, Cai et al. loaded miR-2861 into sticky-end bearing tFNAs and demonstrated that RNase H facilitated unloading of miRs from stFNA-miR [37]. This led to enhanced expression of Runx2 and ALP and promoted bone regeneration at bone defect sites (Fig. 5B). Furthermore, tFNAs have been shown to protect cartilage by promoting autophagy and inhibiting apoptosis of IL-1β-stimulated chondrocytes [38]. These findings highlight the potential of tFNAs as versatile tools for bone and cartilage regeneration. However, the production of tFNAs can be time-consuming and expensive, which may limit their widespread use in clinical settings. Further research is needed to develop scalable and cost-effective production methods.

#### RNA

**Fig. 6:**
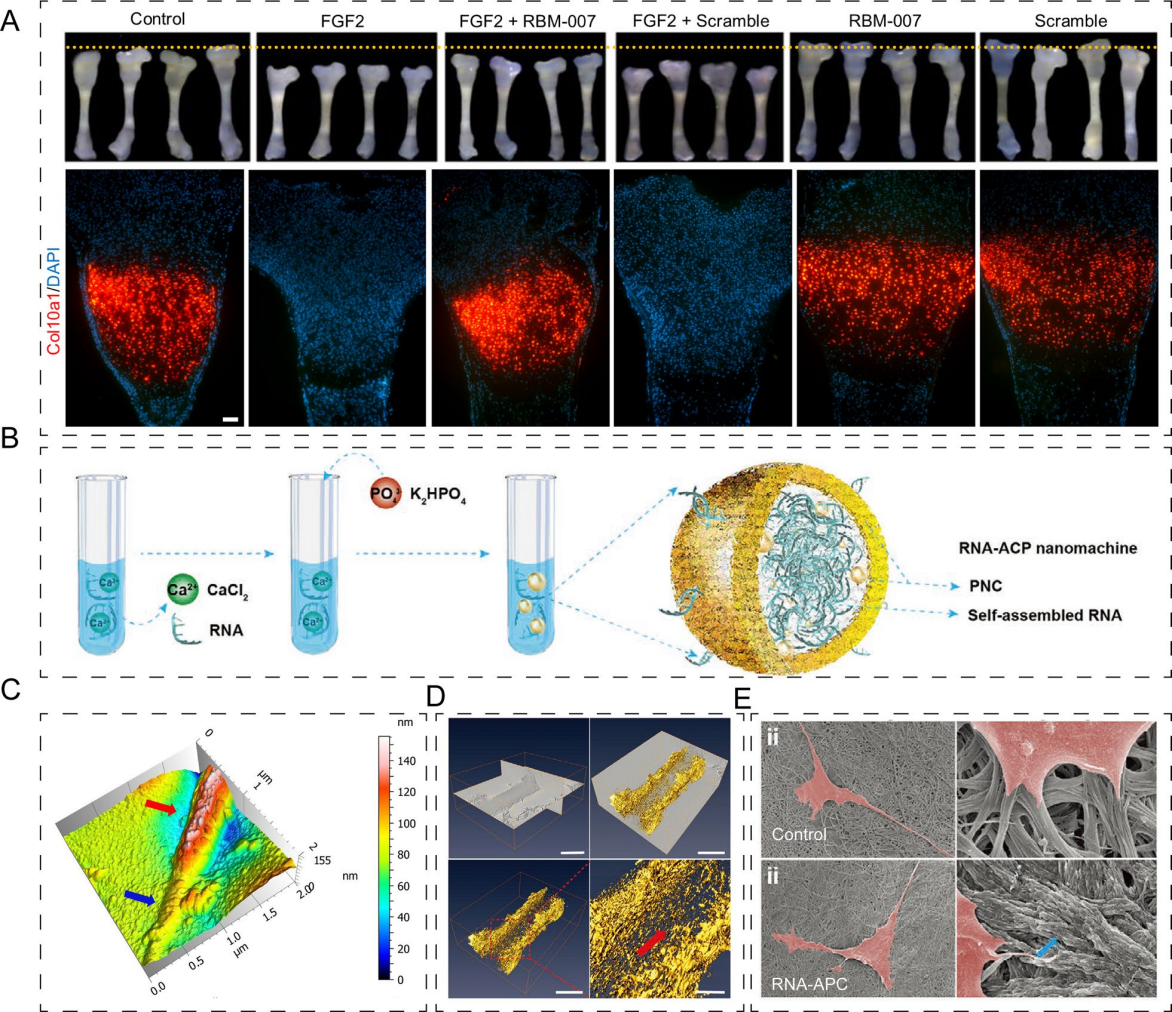
Application of RNA-based composites in bone/cartilage regeneration. **A** Appearance of the E18 mouse embryos tibiae cultured in different medias (upper) and representative immunofluorescence images of col10a1(lower). Reprinted with permission [40]. **B** Schematic illustration of the formation of RNA-ACP nanomachine. **C** AFM images of collagen fibril in RNA-ACP media for 5 h. **D** Cryo-electron-tomography images of intrafibrillar mineralization in RNA-ACP media for 24 h (upper) and 3D visualization of a mineralized fibril (lower). **E** SEM images with different magnifications of BMSCs cultured in control and RNA-ACP media for 1 day. B–E Reprinted with permission [17]

RNA aptamers have recently garnered significant research interest in the field of bone and cartilage regeneration. RBM-007, an RNA aptamer specific to fibroblast growth factor 2 (FGF2), has been identified as a potent inductor of angiogenesis and fibrosis [39, 40]. Pavel Krejci et al. [40] used tibia organ culture and found that RBM-007 inhibited fibroblast growth factor receptor 3 activation by fibroblast growth factor 2 and restored the impaired differentiation of chondrocytes eventually. In addition, aptamer RBM-007 increased the expression of Col10a1(collagen type X alpha 1 chain), but was not appropriate for a scrambled aptamer (Fig. 6A). This suggests that RNA aptamers may have therapeutic potential for skeletal dysplasia. Additionally, RNA aptamers can be applied to treat bone disease pain and bone-marrow aplasia [41]. For instance, Yoshikazu Nakamura et al. generated APT-F2, an RNA aptamer specific for FGF2, to act as an antagonist or inhibitor of FGF2, counteracting its negative effects on bone, such as inhibiting osteoprotegerin (OPG) production, promoting osteoclast differentiation, and stimulating angiogenesis. And found that PEGylated APT-F2 effectively prevented bone disruption in arthritis and osteoporosis [16]. In another study, a CD40 2-fluoro-RNA oligonucleotide aptamer was generated using SELEX. This study showed that treatment with the CD40 agonist aptamers promoted bone-marrow aplasia recovery, as evidenced by enhanced expression of beta-catenin upon treatment with CD40Apt1-dimer agonistic aptamer [16, 42]. These findings highlight the potential role of RNA aptamers as effective therapeutics for various bone-/ cartilage-related disorders. While RNA aptamers are highly specific to their target molecules, there is still the risk of off-target binding, which can lead to unintended effects. Careful selection and validation of the RNA aptamer sequence and target molecule are necessary to minimize the risk of off-target effects.

Meanwhile, RNA molecules have emerged as an hotspot of research in the field of nanomaterials due to their ability to self-assemble into specific nanostructures in response to external stimuli, and carry out specific functions [43, 44]. Previous study has demonstrated that RNA nanomachines can be utilized to promote bone healing by improving extracellular matrix (ECM) mineralization, stabilizing amorphous calcium (APC) phosphate, and promoting new bone regeneration [17]. The RNA-APC composed of: (i) total RNA extracted from mBMSCs, and (ii) supersaturated calcium phosphate solution (Fig. 6B). In vitro studies have demonstrated that RNA-APC effectively induced collagen mineralization and supported the proliferation of mBMSCs, while in vivo studies have revealed its potential as an osteoinductive and osteogenic material for bone regeneration (Fig. 6C–E). These findings highlight the promise of RNA molecules as a new class of materials for bone tissue engineering.

While nucleic acid-based biomaterials hold promise for bone and cartilage regeneration, there are several challenges that need to be addressed for their successful application. For example, nucleic acids can trigger immune responses, leading to inflammation and potential toxicity. Minimizing immunogenicity and ensuring the safety of nucleic acid-based biomaterials is a critical consideration for their use in regenerative medicine. In addition, developing scalable manufacturing processes for nucleic acid-based biomaterials is necessary for their widespread clinical use. Optimizing production methods, ensuring reproducibility, and controlling batch-to-batch variability are important considerations for large-scale production.

### Protein-inspired building blocks

Proteins are complex organic polymers that possess intricate structures. They are composed of amino acids arranged as peptide chains, which can then fold to form proteins with three-dimensional structures [45]. Protein-based scaffolds, integrated with other components such as hydrogel, hydroxyapatite, and chitosan [46–48], have been effectively employed in bone and cartilage regeneration. Meanwhile, owing to their availability, targeting specificity, and small size, amino acids-based peptides have been developed for incorporation into disease treatment materials [49].

#### Collagen

**Fig. 7:**
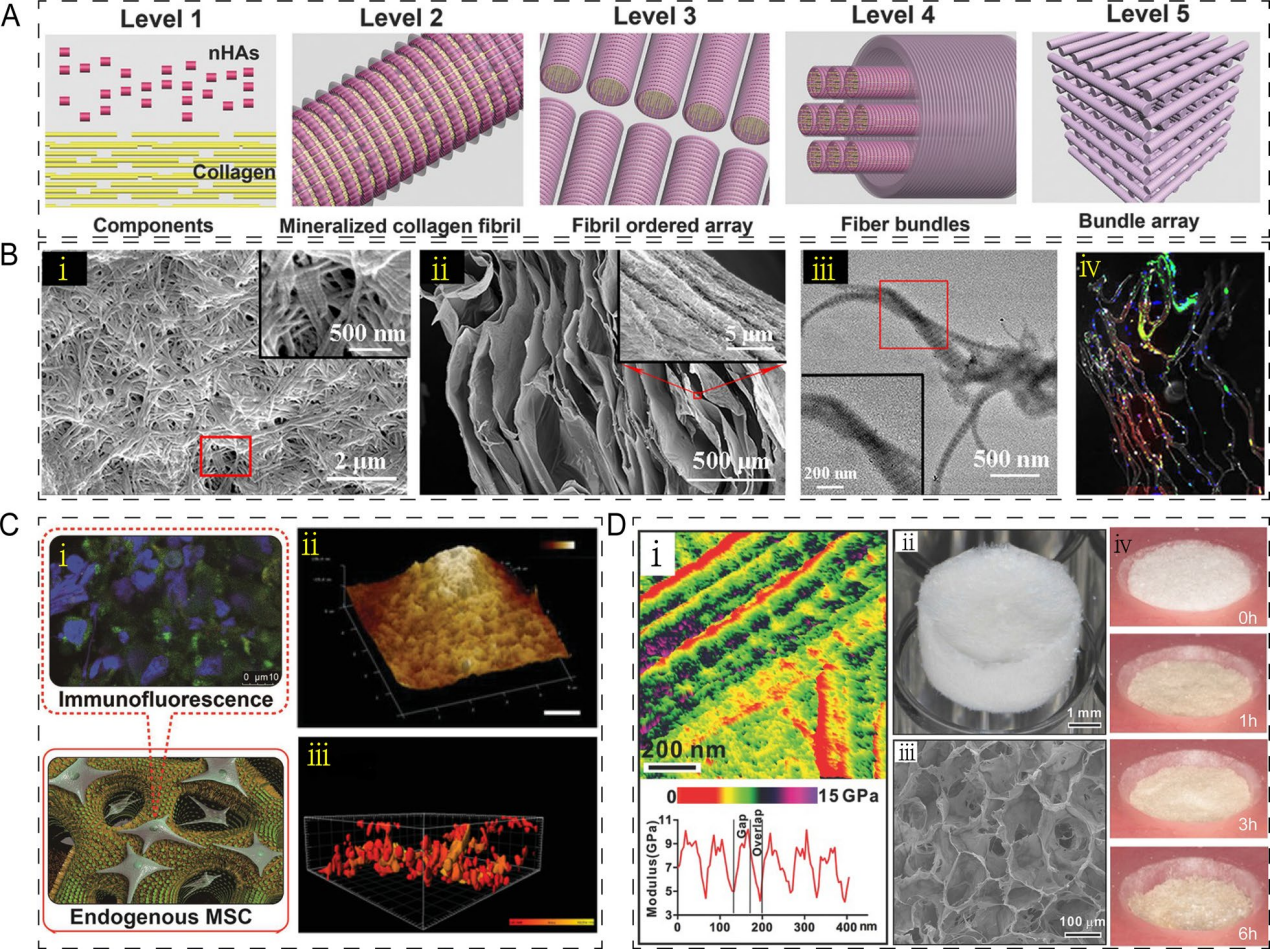
Application of collagen-based composites in bone/cartilage regeneration. **A** Schematic illustration of the process of organization of natural bone. Reprinted with permission [56]. **B** Surface (i) and cross-sectional (ii) SEM morphologies and TEM (iii) images of Col-FeMnHA-lamellar scaffold. And merge image of bone sialoprotein and dentin matrix protein 1 expressing cells in Col-FeMnHA-lamellar (iv). Reprinted with permission [54]. **C** Nanosilica-COL Scaffolds improved endogenous MSCs recruitment and osteogenesis (i). AFM images (ii) and cell surface area 3D rendering images (iii) of tetramethoxysilane nanosilica-COL scaffolds. Reprinted with permission [55]. **D** AFM property maps image (i, upper) of HIMC and analyses of Young's modulus (i, lower). Morphology (ii) and cross section ii representative image of 3D-COL Scaffolds. Morphology changes of HIMC treated by collagenase at 0 h, 1 h, 3 h and 6 h (iv). Reprinted with permission [56]

Collagen (COL) is a major component of the ECM, which shows biodegradability and biocompatibility that has been extensively utilized as a biomaterial for bone and cartilage regeneration [50]. In particular, COL serves as a template for the biomineralization and deposition of calcium phosphate [51]. During the organization of natural bone, mineralized collagen fibrils are at the second level (Fig. 7A). However, pure COL lacks mechanical strength and stiffness when used as a scaffold for bone regeneration. Current and emerging scaffolds prefer combining COL with other components, such as HA, silica, chitosan and hydrogel, to optimize the mechanical properties of COL scaffold [52, 53]. For example, Wei et al. developed the COL-HA-lamellar scaffold, which exhibited greater tensile strength than the COL-HA-cellular scaffold. They then incorporated iron and manganese into the COL-HA-based lamellar scaffold to improve osteoinductivity, as evidenced by in vitro osteogenic differentiation and in vivo bone regeneration ability (Fig. 7B) [54]. In another study, Yu et al. investigated the effects of COL-nanosilica on bone regeneration and found that a fully covered COL scaffold with nanosilica promoted BMSCs recruitment, osteogenesis, and matrix mineralization (Fig. 7C) [55]. A COL/chitosan/biphasic calcium phosphate porous tri-component composite scaffold incorporated with compound K was also prepared by the freeze-drying method, improving mineralization and cell adhesion [46]. Additionally, COL-based composite hydrogels appeared to have higher stiffness and contribute to chondrogenesis [48]. Intrafibrillar mineralized COL created collagenous gap regions that provided a microenvironment for BMSCs to promote bone regeneration. Hierarchical, intrafibrillarly mineralized collagen (HIMC) provided excellent strength similar to natural bone. 3D HIMC scaffolds were assembled with high porosity, interconnected pores, and biodegradability (Fig. 7D). The use of 3D HIMC scaffolds enabled bone regeneration to be achieved in vivo [56].

Due to its economic value and environmental sustainability, marine COL has gradually replaced COL from bovine and porcine tissues [57]. Marine COL scaffolds have also demonstrated desirable effects for bone regeneration. Natural marine sponge COL has been identified as an osteogenesis scaffold due to its collagenous fibrous network [58]. Michael et al. [59] evaluated the parameters of jellyfish COL to find an optimal one for cartilage tissue engineering. The results showed that 3D jellyfish COL scaffolds were proved to promote chondrogenic stimulation of BMSCs. While collagen-based scaffolds have shown promising results in preclinical studies [60], their clinical translation has been limited by factors such as cost, regulatory requirements, and patient variability. Further studies are needed to optimize the clinical protocols, patient selection, and long-term outcomes of collagen-based therapies.

#### Silk fibroin

Silk fibroin (SF) is a widely used protein in natural silk-based biomaterials for bone and cartilage regeneration. SF, extracted from silk of silkworms coated with sericin, is composed of a 26 kDa light chain and a 390 kDa heavy chain [61]. Due to its biocompatibility, biodegradability, hypoimmunity, and remarkable mechanical properties, SF has tremendous potential in functional biomedical materials [62, 63].

**Fig. 8:**
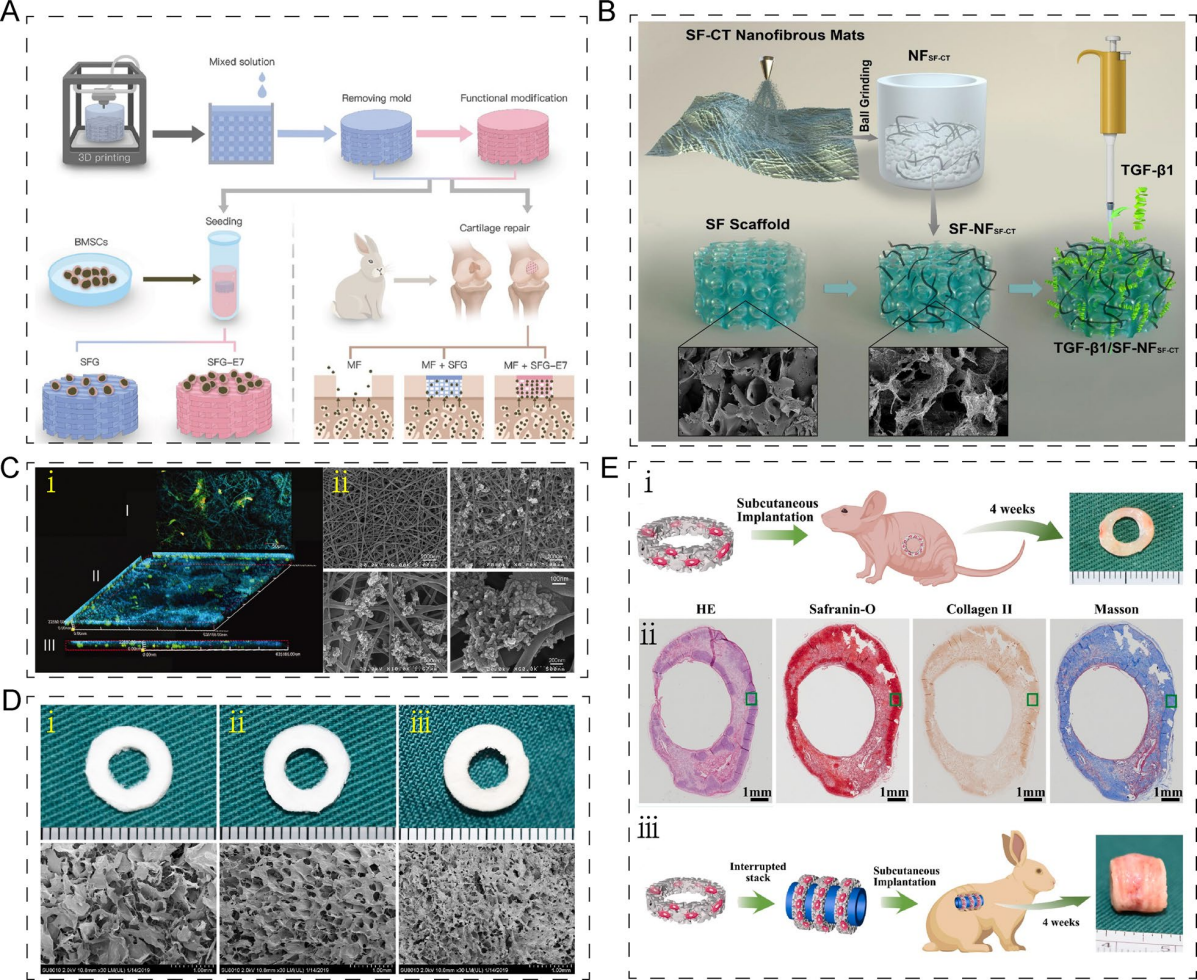
Application of silk fibroin-based composites in bone/cartilage regeneration. **A** Schematic illustration of SF-gelatin scaffold generation and function. Reprinted with permission [64]. **B** Schematic illustration of TGF-β1 into SF-chitin composite scaffold. Reprinted with permission [66]. **C** Schematic illustration of the fabrication of SF/diglycidyl ether hydrogel microspheres and the treatment of OA. Reprinted with permission [67]. **D** Schematic illustration of the fabrication of integral bilayer methacrylated SF hydrogel scaffold and its role of osteochondral repair. Reprinted with permission [68]. **E** Immunofluorescence of 3D cultivation of MC3T3-E1 (i). SEM images of pure SF (ii, upper) and mineralized SF/ nano-HA nanofibrous (ii, lower) with different magnifications. Reprinted with permission [69]. **F** Gross views (upper) and SEM (lower) images of DCM (i), DCM/SF (ii), and SF (iii) scaffolds. Reprinted with permission [70]. **G** Schematic illustration of BMSC-DCM/SF implanted into mice (i). The results of HE, safranin-O, immunohistochemical COL2 and Masson staining of cartilaginous ring (ii). Schematic illustration of bionic trachea generated by BMSC-DCM/SF implanted into rabbit (iii). Reprinted with permission [70]

Specifically, SF has been extensively studied for cartilage and osteochondral repair. One promising SF-gelatin scaffold was generated using 3D printing technology by Ao et al. [64] The scaffold was connected with a BMSC-specific-affinity peptide by co-incubation for 24 h at 4 °C to recruit and retain BMSCs from subchondral bone for in-situ cartilage repair. The uniform 350 µm pore size of the scaffold was suitable for cell proliferation and differentiation by matching the thickness of rabbit articular cartilage (Fig. 8A). Additionally, the composite hydrogel formed by the phenolic hydroxyl groups in propanoic acid-modified chitosan (PC) crosslinked with the tyrosine in SF was injectable and variable in shape, which could accommodate to shapes of cartilage defects [65]. Adding transforming growth factor-β1 (TGF-β1) into SF-chitin composite scaffold promoted cartilage regeneration by recruiting BMSCs (Fig. 8B) [66]. Recently, SF hydrogel microspheres, used as a bio-lubricant, has been extensively studied in the treatment of OA. For instance, David et al. fabricated the SF/diglycidyl ether hydrogel microspheres, where SF have been used for enhanced mechanical and structural stability of hydrogel microspheres. These injectable hydrogel spheres functioned optimally as a bio-lubricant, providing significant pain relief, long residence time, biocompatibility, and cartilage tissue repair capability in the treatment of osteoarthritis. (Fig. 8C) [67]. The same results in another study, Liu et al. demonstrated that integral bilayer methacrylated SF hydrogel scaffold, combined with platelet-rich plasma and the SF-kartogenin and SF-berberine microspheres, accelerated osteochondral repair by enhancing chondrogenic and osteogenic differentiation of BMSCs (Fig. 8D) [68]. SF combined with nano-HA showed higher bone conductivity. SF/nano-HA nanofibrous scaffolds were found to be beneficial for the 3D cultivation of MC3T3-E1 (Fig. 8E) [69]. The trachea is constituted of cartilaginous rings and vascularized fibrous tissue, and chondroinductive activity is essential for trachea tissue regeneration. Besides chondroinductive activity, porous sponge structure gave SF-DCM (decellularized cartilaginous matrix) scaffold eligible mechanical properties. The results of HE (hematoxylin and eosin staining), safranin-O, immunohistochemical COL2 (type II collagen) and Masson staining showed the chondroinductive activity of SF-DCM scaffold carried BMSCs in vivo (Fig. 8F, G) [70].

Furthermore, SF has the potential to promote angiogenesis for significant bone ingrowth [71]. SF nets were found to support the growth and spread of different kinds of cells, especially endothelial cells, which are essential for angiogenesis. The research of Kirkpatrick group showed that SF nets were excellent for activation, maintenance and angiogenic potential of endothelial cells [72]. Moreover, SF scaffolds also promoted pre-vascular structures and osteogenic differentiation through co-culturing osteoblasts and endothelial cells [73]. Here, what we need to note is that silk fibroin can elicit an immune response in some individuals, leading to inflammation or fibrosis. The immunogenicity of silk fibroin needs to be carefully evaluated and minimized through purification, processing, and sterilization methods [74].

#### Antibody

Antibodies, also known as immunoglobulins, are proteins produced by the immune system in response to the presence of foreign substances called antigens [75]. Antibodies bind to specific antigens and help to neutralize or eliminate them from the body.

In the context of bone tissue engineering, antibodies can be used as a biomaterial to target specific cells or molecules involved in bone/cartilage formation or regeneration. Antibody-functionalized microspheres are a promising approach for in situ bone regeneration. These microspheres are typically composed of biocompatible materials, such as chitosan, and are functionalized with antibodies or other targeting molecules that bind specifically to cell surface receptors on stem cells involved in bone formation and repair. For example, Li et al. fabricated a polydopamine coated chitosan microspheres and then functionalized with CD271 antibody, which is an effective factor for BMSCs recruitment. The functional microspheres exhibited excellent efficiency in selectively attracting BMSCs and promoting their attachment and growth, which facilitated the bone formation in vivo [76]. In the context of recruitment of cells, antibodies also could be attached onto other surface of biomaterials. For instance, Chen et al. attached leptin receptor antibody on an electrospun scaffold, which grafted with BMP2 (bone morphogenetic protein-2)-loaded hollow MnO_2_. As a cell surface marker for SSCs, leptin receptor antibody attached onto the surface of the bionic periosteum demonstrated excellent ability to recruit SSCs (skeletal stem cells) in situ [77]. In addition, antibodies also be used for delivering proteins to injury sites. In bone, the biomaterials conjugated with BMP2 antibody could deliver BMP2 to the desired site and realize controlled release manner [78].

Antibodies and aptamers discussed above are both types of biomolecules that can be used as a part of scaffold for targeted delivery in orthopedic applications [79]. However, they differ in their molecular structures, binding properties, and advantages and disadvantages. One of the advantages of aptamers over antibodies is that they are smaller and more stable, and can be produced synthetically, making them easier to modify and optimize for specific applications. Aptamers can also have higher binding affinity and specificity than antibodies and can target a wider range of molecules, including small molecules, toxins, and cell surface receptors. However, antibodies have some advantages over aptamers as well [80]. Antibodies have a longer half-life in the body, allowing for sustained therapeutic effects. They can also be produced in large quantities using mammalian cell culture systems, which is not currently possible with aptamers [81]. In summary, both aptamers and antibodies have their own advantages and disadvantages, and the choice between them depends on the specific application and target.

#### Peptide

As previously discussed, COL and SF based scaffolds have been identified as the optimal biomaterials for bone and cartilage regeneration. In terms of molecular signaling, bone morphogenetic proteins (BMPs) have been found to be the most effective protein for promoting bone formation [82]. However, the use of BMPs can be expensive and may have adverse effects. An alternative approach is the use of peptides, which can be obtained either through engineering or naturally occurring processes [83]. Peptides have been shown to possess a range of effects, including promoting bone regeneration [84], inhibiting bone resorption, promoting cell adhesion, and promoting angiogenesis (Table 1) [84, 85].

**Table 1:** Peptide-candidates for bone/cartilage regeneration

Function	Name	Sequence	Refs.
Inhibiting bone resorption	WP9QY	YCWSQYLCY	[86]
	OP3-4	YCEIEFCYLIR	[87]
	RANKL inhibitor peptide	YCWNSDCECCY	[121]
Promoting bone formation	BFP-1	GQGFSYPYKAVFSTQ	[89]
	BFP-2	VEHDKEFFHPRYHHR	[122]
	CGRP-α	VTHRLAGLLSRSGGVVKNNFVPTN	[123]
	Hexarelin	VGSKAF	[124]
	OGP	HWAWFKALKRQGRTLYGFGG	[90, 125, 126]
	GFOGER	GFOGER	[127]
	KP	KIPK(Ac)ASSVPTELSAISTLYL	[112]
	CATH-2	RFGRFLRKIRRFRPKVTITIQGSARF-NH2	[128]
	DSS	ASS (Asp-Ser-Ser)	[129]
Inhibiting bone resorption and promoting bone formation	NBD	drqikiwfqnrrmkwkk TALDWSWLQTE	
	CGRP	ACDTATCVTHRLAGLLSRSGGVVKNNFVPTNVGSKAF-	[92–94]
	W9	YCWSQYLCY	[95] [96]
Targeting:			
HA	Poly-Asp	(Asp)n	[98]
BMSC	DPI	DPIYALSWSGMA	[99]
Osteoblast	PP102	YRAPWPP	[100]
Exosome	CP05	CRHSQMTVTSRL	[97]
Chondrocyte	CAP	DWRVIIPPRPSA	[101]
Promoting cell adhesion	RGD	AGA (Arg-Gly-Asp)	[108]
	F105	YKRSRYT	[130]
	F36	PDGRVD	[131]
	F77	KEDGRLL	[131]
	PHSRN	PHSRN	[132]
Promoting angiogenesis	QK	KLTWQELYQLK(Ac)YK(Ac)GI (CVRKIEIVRKK)2-Ahx-Ahx-Ahx	[112]
	PBA2-1c	RKRKLERIAR	[133]
	Exendin-4	HGEGTFTSDLSKQMEEEAVRLFIEWLKNGGPSSGAPPPS	[134]
	TP508	AGYKPDEGKRGDACEGDSGGPFV	[135]
Cathelicidin	LL-37	LLGDFFRKSKEKIGKEFKRIVQRIKDFLRNLVPRTES	[113]
	KR-12	KRIVQRIKDFLR	[136]

**Fig. 9:**
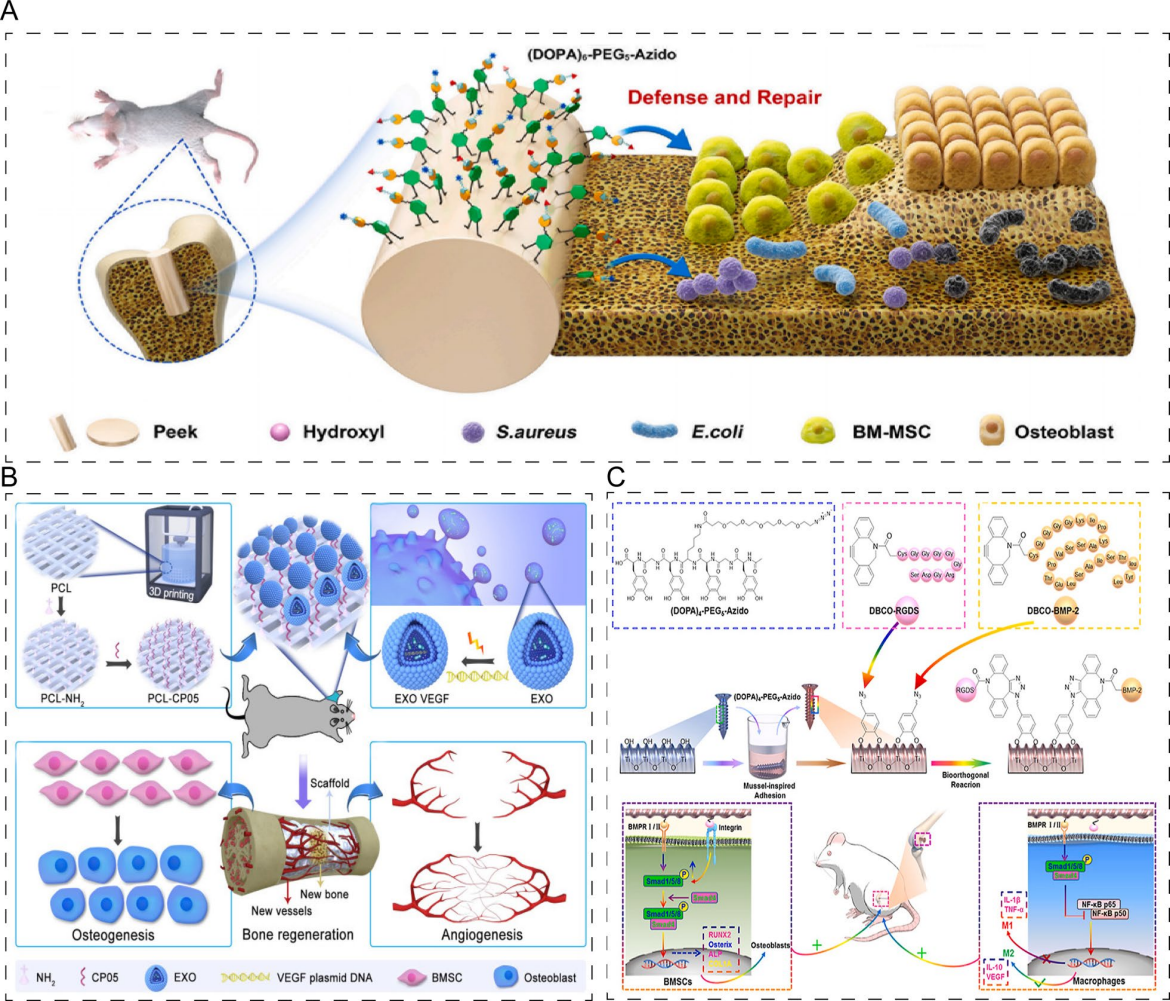
Application of peptides in bone/cartilage regeneration. **A** The schematic diagram of integration of antimicrobial peptide and OGP onto polyetheretherketone surfaces showing anti-infectivity and osteo-inductivity. Reprinted with permission [91]. **B** The schematic diagram of exosome-activated bone scaffolds increased vascularized osteogenesis in vivo. Reprinted with permission [103]. **C** BMP-2 and RGD peptides modified Ti-based implants could enhance BMSCs adhesion and regulate macrophage polarization in rat. Reprinted with permission [111]

For instance, Roland et al. demonstrated that the WP9QY peptide can mimic a TNF receptor (TNFR) ligand and inhibit TNF-α-induced activity by binding to RANK ligand (RANKL) [86]. Moreover, the use of WP9QY peptide prevented bone loss in vivo. Another bone resorption inhibitor, the osteoprotegerin-like peptidomimetic (OP3-4), prevented the interaction between RANKL and RANK by resembling osteoprotegerin. TRAP-positive staining and resorption pits showed that OP3-4 inhibited osteoclast formation and bone resorption [87]. Bone-forming peptide-1 (BFP-1), a derivative of bone morphogenetic protein-7 (BMP-7), has been shown to promote bone formation [88]. In a study conducted by the group of Shicheng Wei, BFP-1 was stored in mesoporous silica nanoparticles (MSNs) and encapsulated in alginate hydrogel treated with RGD to obtain pep@MSNs-RA. RGD peptides are integrin binding ligands that promote the proliferation and adhesion of MSCs [89]. This time-responsive system functions in a manner similar to ECM and facilitates the survival and growth of MSCs [89]. Osteogenic growth peptide (OGP), which is derived from bone marrow and promotes osteogenesis and hematopoietic function, has been functionalized with tetrahedral framework nucleic acids (tFNAs) to improve the structural stability of tFNAs and deliver OGP more efficiently [90]. In addition, Geng et al. have integrated an antimicrobial peptide and OGP onto polyetheretherketone surfaces using a biomimetic surface strategy that demonstrates antibacterial effects and provides sufficient osteogenic activity, as indicated by the results in vivo and vitro (Fig. 9A) [91]. Furthermore, peptides such as NBD [92–94], CGRP [95] and W9 [96] exhibit a dual effect of inhibiting bone resorption and promoting bone formation.

The most important function of peptides is targeting, such as HA, BMSCs, osteoblasts, exosomes and chondrocytes [97–101]. Acidic oligopeptides, comprising of acidic amino acids such as Aspartic acid (Asp) and Glutamic acid (Glu), are widely used as bone-targeting carriers by binding to HA [102]. Gao et al. have developed an exosome targeting peptide (CP05) by conjugating it with CD63, a protein present on the surface of exosomes [97]. Given the exosomal anchor property of CP05, Wang et al. modified the bone scaffolds with CP05 to capture engineered ATDC5-derived exosomes carrying VEGF plasmid [103]. The results showed that exosome-activated bone scaffolds increased vascularized osteogenesis in vivo (Fig. 9B). Another example is the chondrocyte-affinity peptide (CAP), which was identified using phage display technology and has been widely used for chondrocyte-specific delivery [104]. For instance, hybrid exosomes containing surface-displayed CAP fused with liposomes were developed as a genome editing tool, which effectively alleviated osteoarthritis by targeting MMP-13 in chondrocytes in vivo [105]. Engineered chondrocyte-specific exosomes have also been utilized for the delivery of miR-140, a protective miRNA for chondrocytes, in the treatment of osteoarthritis [106].

Arg-Gly-Asp (RGD) peptides were identified as adhesion sequences and identified to promote cell adhesion and differentiation by binding to integrins. To enhance bone and cartilage regeneration, diverse biomaterials including liposomes, polycaprolactone [107], and poly(lactic-co-glycolic acid) (PLGA) have been modified with RGD (arginine-glycine-aspartic acid) coatings [108–110]. Qin et al. demonstrated that BMP-2 and RGD peptides modified Ti-based implants could enhance BMSCs adhesion and regulate macrophage polarization in rat (Fig. 9C) [111]. In addition, peptides have been shown to promote angiogenesis and prevent bacterial infections. For example, Wang et al. loaded KP and QK peptides to self-healing hydrogel, which realized vascularized bone regeneration by injecting the peptide-hydrogel into rat calvaria [112]. The LL-37 peptide, found in humans, plays a vital role in the immune response and has been demonstrated to enhance BMSCs migration and bone formation in vivo when modified and applied to titanium substrates [113]. While peptides have shown great potential as biomaterials for bone and cartilage regeneration, there are several challenges that need to be addressed to optimize their performance, safety, and clinical translation. The long-term safety and biocompatibility of peptide-based materials need to be evaluated in preclinical and clinical studies. The potential risks and adverse effects of peptide-based therapies need to be carefully monitored and managed to ensure patient safety and ethical use of the materials.

Indeed, some insect proteins have shown promising properties in promoting bone cell proliferation, differentiation, and the regeneration of bone tissue. These proteins possess biological activity and can mimic the functions of growth factors, which are essential for bone development and repair. Moreover, insect proteins contain specific amino acid sequences and domains that can interact with cell surface receptors and activate intracellular signaling pathways involved in bone formation [114]. These proteins, such as royal jelly proteins, can stimulate the expression of osteogenic markers and induce the differentiation of mesenchymal stem cells into osteoblasts, which are responsible for bone synthesis [115]. However, despite their potential, the use of insect proteins in bone tissue engineering is still in its early stages, and further research is needed to explore their full capabilities. Factors such as scalability, purification methods, and long-term stability must be considered to ensure the practical application of these proteins in clinical settings.

Supramolecular peptides are peptides that are designed to self-assemble into larger, more complex structures through non-covalent interactions such as hydrogen bonding, van der Waals forces, and electrostatic interactions [116]. These structures can range in size from small nanospheres to large hydrogels and can have a variety of functions, such as drug delivery, tissue engineering, and sensing. Supramolecular peptides have been investigated as potential biomaterials for bone and cartilage regeneration due to their ability to self-assemble into nanofibrous networks that mimic the extracellular matrix (ECM) of these tissues [117]. The nanofibrous network can provide a 3D scaffold that promotes cell adhesion, migration, proliferation, and differentiation [74].

One type of supramolecular peptide that has been studied extensively for bone/cartilage regeneration is the peptide hydrogel. These hydrogels can be synthesized from self-assembling peptides that contain a hydrophobic core and hydrophilic surface groups. The hydrophobic core drives the self-assembly of the peptides into a 3D network, while the hydrophilic surface groups interact with water molecules to form a hydrated gel. Studies have shown that peptide hydrogels can support the growth and differentiation of bone and cartilage cells in vitro and in vivo. For example, Wu et al. conducted studies using a RADA16 scaffold for co-culturing osteogenic adipose-derived stem cells (ADSCs) and endothelial ADSCs. The results demonstrated that the cells exhibited strong adhesion to the RADA16 scaffold, leading to enhanced osteogenesis and angiogenesis. This was attributed to the excellent biocompatibility of the RADA16 scaffold, which effectively promoted cellular biological activity [118]. Similarly, Wang et al. demonstrated that a peptide hydrogel composed of the self-assembling peptide RAD16-I and the growth factor TGF-β1 was found to enhance the chondrogenic differentiation of MSCs and promote cartilage repair in a rabbit model of osteochondral defect [119]. In addition to peptide hydrogels, supramolecular peptides have also been investigated as coatings for orthopedic implants to promote bone and cartilage regeneration. For example, self-assembling peptide hydrogels have been demonstrated to promote chondrogenesis more effectively than agarose, as evidenced by their ability to increase extracellular matrix production, enhance DNA content, and improve the molecular structure of aggrecan [120].

Overall, supramolecular peptides have shown great potential as biomaterials for bone and cartilage regeneration due to their ability to mimic the natural ECM of these tissues and promote cell adhesion, proliferation, and differentiation. Further research is needed to optimize the design and fabrication of these materials and to evaluate their long-term safety and efficacy in clinical settings.

### Lipid-inspired building blocks

lipids have gained considerable attention as versatile and biocompatible materials due to their amphiphilic nature, which allows them to form a variety of structures with both hydrophobic and hydrophilic domains [137]. Structurally, lipids consist of a polar head, a hydrophobic tail, and a linker between them. Based on their chemical properties, lipids can be categorized into cationic lipids, ionizable lipids, and others, such as phospholipids, cholesterol, or polyethylene glycol (PEG). Cationic lipids contain positively charged head groups, while ionizable lipids can be protonated at low pH [138]. Liposomes, which are spherical vesicles made of phospholipid bilayers, have been widely studied as potential drug delivery vehicles due to their ability to encapsulate hydrophilic and hydrophobic drugs. Additionally, lipid nanoparticles have been explored as promising nanocarriers for the delivery of drugs and diagnostic compounds due to their excellent biocompatibility and low toxicity [139].

**Fig. 10:**
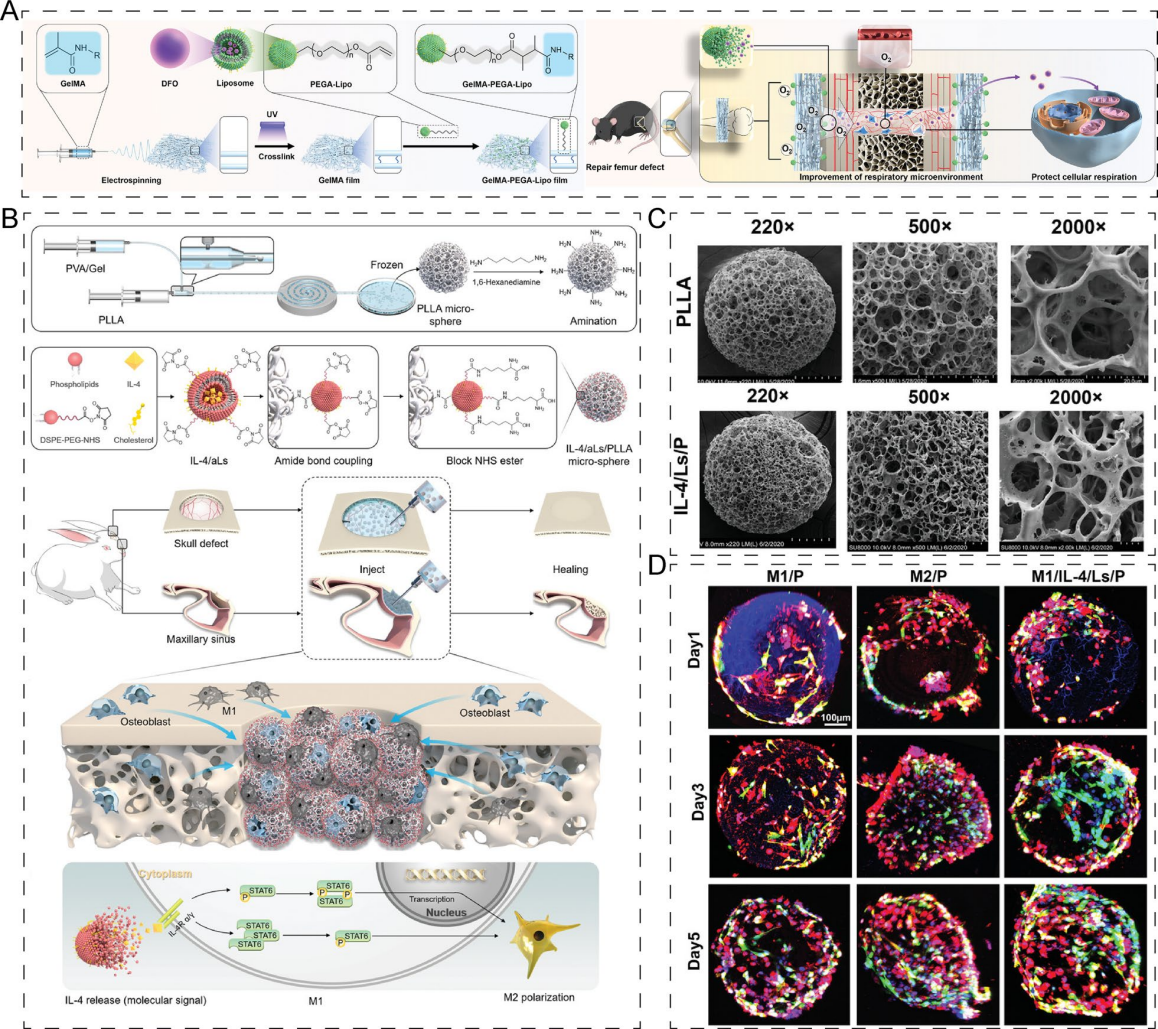
Application of liposome-based composites in bone/cartilage regeneration. **A** Schematic diagram of the fabrication of DFO@Scaffold and its treatment for femur defect. Reprinted with permission [140]. **B** The generation of IL-4/aLs/PLLA and its role in maintaining the phenotype of M2 macrophages, enhancing the differentiation and cell viability of osteoblasts. Reprinted with permission [141]. **C** Scanning electron microscope images of PLLA and IL-4/aLs/PLLA. Reprinted with permission [141]. **D** Confocal images of polarized macrophages (red), MC3T3-E1 (GFP) cultured on microspheres. Reprinted with permission [141]

Due to their exceptional biocompatibility, passive targeting ability, and ability to deliver both hydrophilic and hydrophobic molecules, liposomes and lipid nanoparticles are commonly used as carriers for bone diseases. For example, Chen et al. [140] encapsulated DFO, a Hif-1 pathway activator, into grafted polyethylene glycol acrylate (PEGA) liposomes and loaded DFO@PEGA-Lipo with electrospun fibers of GelMA to form DFO@Scaffold. DFO@Scaffold enhanced the biomechanical properties of the periosteum and protected mitochondrial function by activating Hif-1 in BMSCs (Fig. 10A). Additionally, promoting the transformation of macrophages from immunosuppressive M2 phenotype to pro-inflammatory M1 is a popular technique for bone regeneration. Yu et al. [141] created N-hydroxysuccinimide-modified IL-4 loaded liposomes and coupled them onto microspheres. The findings showed that the microsphere scaffold could sustain the phenotype of M2 macrophages, as well as enhance the differentiation and cell viability of osteoblasts (Fig. 10B–D).

Maintaining low friction is essential to ensure the proper function of synovial joints, and impaired lubrication can lead to severe friction-related diseases, such as osteoarthritis. To address this issue, Jacob Klein et al. [142] reported that certain phosphatidylcholine liposomes can act as efficient boundary lubricants. Specifically, when hydrogenated soy phosphatidylcholine (HPSC) lipids were adsorbed onto sliding surfaces, they self-assembled and reduced the coefficient of friction (μ) down to values of μ ≈ 10^–4^ − 2 × 10^–5^, indicating remarkably low friction. This study suggested that liposomes possess lubricating properties, which is further supported by the fact that phospholipids are ubiquitous molecules in synovial joints and can act as lubricants for articular cartilage together with hyaluronan and lubricin [143]. Furthermore, Huang et al. [144] developed a method to incorporate rapamycin (RAPA) into HPSC liposomes and integrated them into a methacrylated hyaluronic acid matrix (Lipo@HMs) using microfluidic technology and photopolymerization. The results showed that liposomes were exposed after friction compared with newly prepared Lipo@HMs, which indicated the function of liposomes in forming a self-renewable hydration layer. The use of RAPA@Lipo@HMs alleviated OA progression in vivo, as evidenced by the results of μCT. However, there are several challenges associated with the application of liposomes as boundary lubricants in bone. For instance, liposomes need to be stable and durable in the biological environment to ensure long-term effectiveness as boundary lubricants. They need to resist degradation, oxidation, or aggregation, and maintain their lubricating properties under various physiological conditions. What’s more, liposomes need to be biodegradable and efficiently cleared from the body to avoid toxicity and immune reactions. The biodegradation and clearance of liposomes need to be carefully studied and optimized to ensure their safety and effectiveness.

### Polysaccharide-inspired building blocks

Polysaccharides are a class of biomacromolecules that have gained increasing interest in the field of biomaterials due to their biocompatibility, immunoactivity, and chemical modifiability. It is well known that polysaccharides are polymers and linked by at least 10 monosaccharides through glycosidic bonds [145]. In this section, we classify polysaccharides into three groups based on their charge: neutral polysaccharides, polyanion polysaccharides, and polycation polysaccharides. Scaffolds based on different charged polysaccharides have shown promise for regenerative applications [146]. The choice of polysaccharide for bone applications should consider factors such as charge, molecular weight, degree of cross-linking, porosity, biodegradability, and biocompatibility. The optimal combination of these factors will depend on the specific requirements of the application, such as the size and shape of the defect, the desired rate of tissue regeneration, and the need for controlled drug delivery. And when using polysaccharides in scaffolds for bone applications, it is important to consider factors such as composition, source, processing method, incorporation of bioactive molecules, and in vivo environment. By optimizing these factors, it may be possible to create scaffolds that are better able to support bone/cartilage regeneration.

#### Neutral polysaccharide

**Fig. 11:**
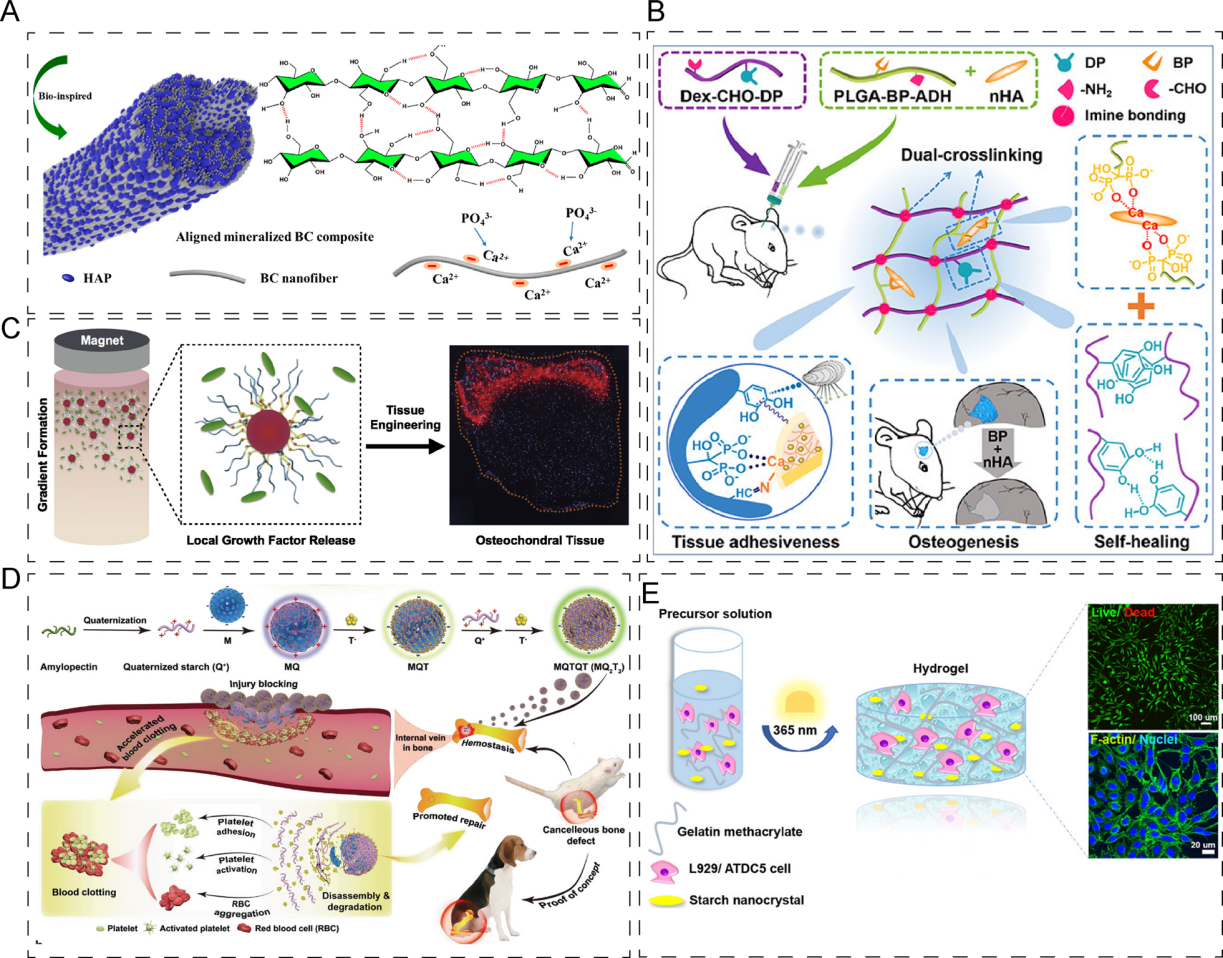
Application of neutral polysaccharide-based composites in bone/cartilage regeneration. **A** CaCl_2_ and K_2_HPO_4_ solutions to mineralize the aligned BC and incorporated HA into aligned and mineralized bacterial cellulose. Reprinted with permission [147]. **B** Aldehyde–catechol-difunctionalized dextran endowed injectable hydrogels with tissue adhesiveness and self-healing ability. Reprinted with permission [151]. **C** Glycosylated superparamagnetic nanoparticle pre-loaded with continuous-gradients BMP-2 showed exciting mineralization ability. Reprinted with permission [153]. **D** Biomass‐derived multilayer‐structured microparticles which were constituted by starches and plant polyphenols to control sustained bleeding and accelerate bone repair in vivo. Reprinted with permission [154]. **E** 3D nanocomposite hydrogels with starch nanocrystal enhanced the proliferation of chondrogenic. Reprinted with permission [155]

Cellulose is a linear polysaccharide composed of hundreds of d-glucose units. Due to its renewable and biodegradable nature, cellulose-based scaffolds have been widely used in bone tissue engineering [147]. Of particular interest is bacterial cellulose (BC), which exhibits high purity and crystallinity, and mechanical properties similar to those of bone tissues. Inspired by natural bone, Zhu et al. mineralized aligned BC with CaCl_2_ and K_2_HPO_4_ solutions, and incorporated HA to create aligned and mineralized bacterial cellulose scaffolds with high mechanical strength and good osteoconductivity (Fig. 11A) [147]. Meanwhile, plant-extracted cellulose scaffolds functionalized with chemical oxidation and surface modification demonstrated excellent proliferation and differentiation of osteoblasts, and in rats, active angiogenesis was observed [148]. Furthermore, incorporating cellulose nanoparticles into chitosan/silk fibroin scaffolds was found to induce significant M1 to M2 macrophage polarization and regulate osteo-immunomodulatory responses [149].

Dextran is a linear polysaccharide consisting of d-glucose backbones linked by α-(1 → 6) bonds [150]. Dextran-based composites have shown significant potential for biomedical applications in bone tissue engineering. For instance, injectable hydrogels endowed with tissue adhesiveness and self-healing ability were developed by aldehyde-catechol-difunctionalized dextran. The hydrogels, coupled with bisphosphonates, were found to promote bone repair significantly by enhancing the process of osteogenesis (Fig. 11B) [151]. Lobat Tayebi fabricated dextran hydrogels using chemical crosslinking reaction incorporated with bioactive glass–ceramic which showed enhanced swelling capacity. The results demonstrated that the composite hydrogel scaffolds were able to support human osteoblast attachment, growth, and proliferation, indicating their promising use for bone tissue engineering [152].

Agarose is a neutral linear polysaccharide widely used in cell culture due to its gelation properties. For instance, glycosylated superparamagnetic nanoparticles pre-loaded with continuous-gradients growth factors, such as bone morphogenetic protein 2 (BMP-2), have shown exciting mineralization ability during osteochondral tissue engineering. The results of quantitative polymerase chain reaction (qPCR) demonstrated significant upregulation of chondrogenic genes and osteogenic genes in osteochondral tissue gradient constructs. Additionally, immunofluorescence (IF) results suggested that osteopontin was present in gradient constructs (Fig. 11C) [153].

Starch is a ubiquitous energy storage polysaccharide that can be extracted from a variety of sources, including higher plants, protozoa, algae, and bacteria. Due to its biocompatibility and low cost, starch has gained increasing attention as a promising biomacromolecule for the development of biomaterials in bone and cartilage tissue engineering. Xu et al. [154] recently developed multilayer-structured microparticles composed of starch and plant polyphenols to control sustained bleeding and accelerate bone repair (Fig. 11D). Micro-CT (micro-computed tomography) results showed that these microparticles promoted the volume of periosteal callus more significantly than the control groups due to the bleeding treatment. Moreover, starch nanocrystals have also attracted increasing attention for their potential applications in bone and cartilage tissue engineering. For example, researchers have incorporated starch nanocrystals into 3D nanocomposite hydrogels to enhance the compressive modulus, leading to increased proliferation of chondrogenic cells in these hydrogels (Fig. 11E) [155].

#### Polyanion polysaccharide

**Fig. 12:**
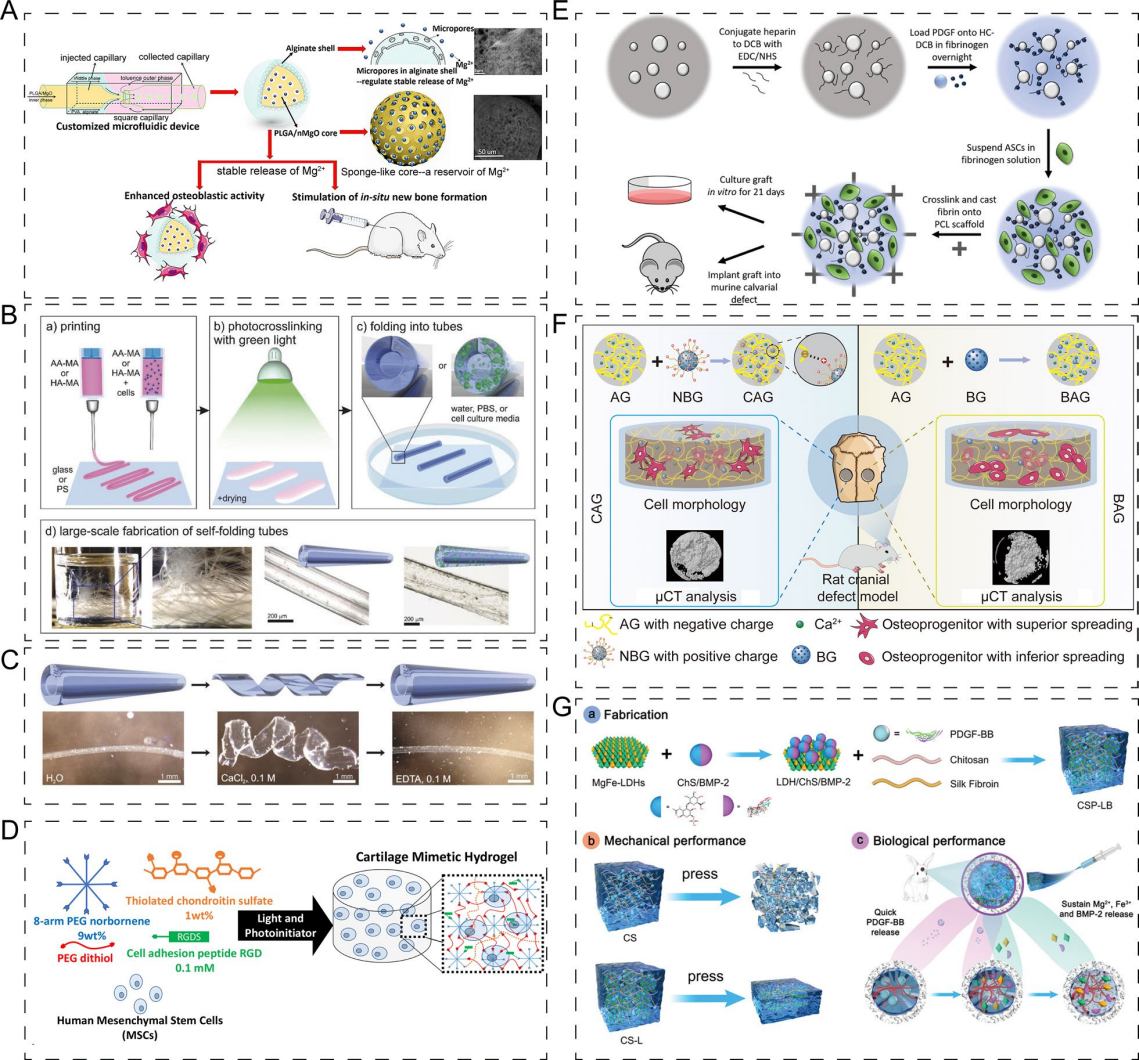
Application of polyanion/ polycation polysaccharide-based composites in bone/cartilage regeneration. **A** Choosing alginate as the shell structure for PLGA/MgO-alginate core–shell microsphere, and the magnesium ions released in situ enhanced osteoblastic activity and promoted new bone formation. Reprinted with permission [158]. **B**, **C** Schematic illustration of generation of 4D self-folding hydrogel-based tubes (**B**), and its responsiveness (**C**). Reprinted with permission [159]. **D** Schematic illustration of cartilage-mimetic hydrogel, which supported a stable chondrogenic phenotype. Reprinted with permission [163]. **E** Generation of heparin-conjugated and decellularized bone particles, and the experiences in vitro and vivo. Reprinted with permission [166]. **F** Schematic illustration of biopolymer hydrogel in which AG and NBG were used to form electrostatic interactions, and the enhanced bone regeneration by biopolymer hydrogel. Reprinted with permission [168]. **G** Schematic illustration of generation of thermal-responsive chitosan/silk fibroin hydrogel, which had good mechanical properties and enhanced the biological properties. Reprinted with permission [173]

Alginate is a linear polysaccharide that exhibits biocompatibility and biodegradability, but its lack of sufficient mechanical properties and long-term stability usually necessitates its incorporation into hydrogels [156]. Crosslinked alginate hydrogels are commonly employed for 3D cell culture. For instance, Mooney et al. [157] developed alginate hydrogels and modulated the nanoscale architecture to enhance the culture properties of MSCs. Osteogenic differentiation was predominantly observed at initial moduli of 11–30 kPa, while adipogenic differentiation was observed when the initial elastic modulus was approximately 9 kPa. In addition to in vitro cell culture, alginate has also been used for bone regeneration. For example, researchers utilized alginate as the shell structure for PLGA/MgO-alginate core–shell microspheres, and the in-situ release of magnesium ions promoted new bone formation (Fig. 12A) [158].

Hyaluronic acid (HA) is a non-sulfated linear polyanion mucopolysaccharide that exists widely in the human body and plays an important role as a component of the extracellular matrix (ECM). Recently, HA has been utilized in 4D bio-fabrication, which enables the generation of constructs that accurately mimic native tissues. One example is the work of Leonid Ionov et al. who used alginate and hyaluronic acid hydrogels as a bioink to create hollow self-folding hydrogel-based tubes that mimic natural vascularized constructs or tubular structures in cortical bone and osteon. This method can also be used to print cell-laden hydrogels (mouse bone marrow stromal cells) that can shape-shift into different structures (Fig. 12B, C) [159]. In cartilage tissue engineering, HA plays a crucial role due to its unique properties. HA possesses excellent biocompatibility, biodegradability, and viscoelasticity, making it an ideal candidate for scaffolds and drug delivery systems [160]. For instance, a HA/RGD pectin hydrogel that incorporates G4RGDS oligopeptide has been shown to provide a host tissue-mimetic microenvironment that maintains chondrocyte phenotype and enhances chondrogenesis [161]. In addition, Wang et al. [162] focused on the modification of hyaluronic acid hydrogels by introducing chemical functional groups to enhance their adhesion to host tissues. The researchers validated the potential application of modified hydrogels in cartilage regeneration through experimental investigations. They employed various anchoring mechanisms to prepare injectable adhesive hydrogels and evaluated their adhesion strength and stability. The experimental results demonstrated that the modified hydrogels could effectively adhere to host tissues and improve the success rate of cartilage regeneration.

Chondroitin sulfate (CS) is an important regulator in cartilage tissue engineering. Incorporating CS and RGD into a PEG hydrogel has been shown to support a stable chondrogenic phenotype while inhibiting MSCs hypertrophy under loading conditions (Fig. 12D) [163]. CS/poly (γ-glutamic acid) hydrogel has also been demonstrated as a potential scaffold for inducing BMSCs differentiation and supporting 3D cell culture and cartilage repair [164]. Furthermore, SC, the most abundant glycosaminoglycan in the body, possesses high levels of hydration. ECM hydrogel functionalized with CS has shown great potential for intervertebral disc regeneration [165].

Heparin is a glycosaminoglycan composed of N-acetylglucosamine, d-glucuronic acid, and L-iduronic acid. It is highly sulfated and is an essential component of the ECM. In clinical settings, heparin is primarily used as an anticoagulant. Grayson et al. [166] utilized heparin-conjugated and decellularized bone particles to create a physiological environment for growth factor presentation. In vitro and in vivo results showed that the bone particles enhanced stem cell osteogenic differentiation and bone formation mediated by bone marrow- or adipose-derived stem cells (Fig. 12E). In addition, Maria-Pau Ginebra et al. [167] applied heparin for osteo-immunomodulation. They found that heparinized beta tricalcium phosphate inhibited the proinflammatory cytokines and enhanced the adhesion and proliferation of MSCs.

Gellan gum (GG) is a polysaccharide extracted from the bacterium *Sphingomonas pancimobilis*. Lai et al. [168] developed a biopolymer hydrogel using alginate/gellan gum (AG) and amino-modified BG (NBG), which formed electrostatic interactions. The results obtained using μCT showed that the biopolymer hydrogel could enhance bone regeneration by improving interfacial compatibility (Fig. 12F). Innovative technologies must be explored to fabricate polysaccharide-based biomaterials. For example, Oreffo et al. [169] combined nanoclay with GG to generate a printable hydrogel. The nanocomposite bioink demonstrated an enhanced printing resolution, which effectively supported cell proliferation and functionality. This improvement was achieved by increasing the printing resolution of the GG hydrogel and providing sustained support for cell proliferation and functionality.

#### Polycation polysaccharide

In biomedical engineering, natural cationic polysaccharides such as chitin or chitosan are commonly used. Chitin is composed of N-acetyl-glucosamine and N-glucosamine units and extracted from crustacean shells. On the other hand, chitosan is a linear polysaccharide and a derivative of chitin, primarily composed of β-(1,4)-linked N-acetyl-d-glucosamine backbones [170]. Due to its high production rate, biodegradability, and hemostatic activity, chitin/chitosan has become a desirable biomaterial for biomedical applications. However, conventional chitin/chitosan hydrogels often exhibit poor bonding properties, leading to increasing interest in modification strategies. For instance, hydroxypropyl chitin with thermosensitivity has been shown to have excellent biocompatibility, biodegradability, and mechanical properties. Jun Xiao et al. [171] demonstrated that thermosensitive hydroxypropyl chitin hydrogel encapsulated with MSCs in a 3D scaffold could promote vascularization and osteoinduction.

The zeolitic imidazolate framework-8 (ZIF-8) nanoparticle was employed to modify catechol-functionalized chitosan hydrogel, and this bone adhesive system exhibited a remarkable capability in promoting vascularized bone remodeling and accelerating the healing of bone wounds in rats [172]. Chitosan, as a versatile biopolymer, has been widely used in the fabrication of hydrogel scaffolds for delivering growth factors. Weng et al. [173] developed an injectable thermal-responsive hydrogel scaffold by incorporating Mg-Fe layered double hydroxide into a chitosan/silk fibroin blend. The resulting hydrogel exhibited excellent mechanical properties, shortened gelation time, and lowered the sol–gel transition temperature. Both in vitro and in vivo experiments showed that the hydrogel scaffold possessed desirable angiogenic and osteogenic properties, leading to enhanced bone regeneration (Fig. 12G).

In addition to hydrogel scaffolds, chitin nanofibers have been shown to have osteoinductive effects. When combined with cell membrane mimetic poly layers, chitin nanofibers promoted new bone's mechanical properties while inhibiting fibroblast growth [174]. To impart osteophilic properties, Prof. Hammond et al. [175] incorporated chitosan /hydroxyapatite nanoparticles into non-degradable electrostatic multilayers containing rhBMP-2. In the osteophilic multilayer, chitosan provided a net positive charge to the macromolecule for the next electrostatic film assembly. The results demonstrated enhanced osteogenic markers and a higher rate of MSC differentiation by the osteophilic multilayer compared to a control substrate.

In summary, polysaccharide-based scaffolds have shown great potential for bone tissue engineering applications. One major advantage of these scaffolds is their biocompatibility and ability to support cell growth and differentiation. Additionally, the renewable and biodegradable nature of polysaccharides makes them a sustainable and eco-friendly option for tissue engineering. However, there are also some potential disadvantages associated with the use of polysaccharide-based scaffolds for bone tissue engineering. For example, neutral polysaccharide-based scaffolds, such as cellulose and dextran, provide good mechanical support and are biocompatible [176]. They are also widely available and cost-effective. However, their lack of bioactivity limits their ability to induce specific cellular responses and may require modifications or the incorporation of bioactive molecules to enhance their efficacy. Examples of polyanionic polysaccharides including hyaluronic acid and alginate, their weak mechanical properties and rapid degradation may limit their use in load-bearing applications [177]. Polycationic polysaccharide-based scaffolds, such as chitosan, have good mechanical strength, biocompatibility, and osteogenesis properties. However, their positive charge may hinder cell adhesion and proliferation, and they may require further modifications to improve their bioactivity. Overall, each type of polysaccharide-based scaffold has its unique advantages and disadvantages. Careful consideration of these factors is necessary when designing polysaccharide-based scaffolds for bone/cartilage tissue regeneration applications.

### Membrane-inspired building blocks

#### Cell membrane

The cellular membrane is predominantly comprised of natural lipid bilayers that are embedded with carbohydrates and proteins. These membranes perform vital biological functions such as cell recognition, signal transduction, and transport of materials while also providing a barrier. Although nanoparticles (NPs) have been used extensively in drug delivery, their clinical use has been limited due to their rapid clearance by the reticuloendothelial system and the circulatory environment [178]. Therefore, an effective drug delivery system must overcome these challenges and target specific cells or tissues. Natural cell membranes have emerged as excellent carriers for NPs as they express specific markers and prevent clearance [179].

**Fig. 13:**
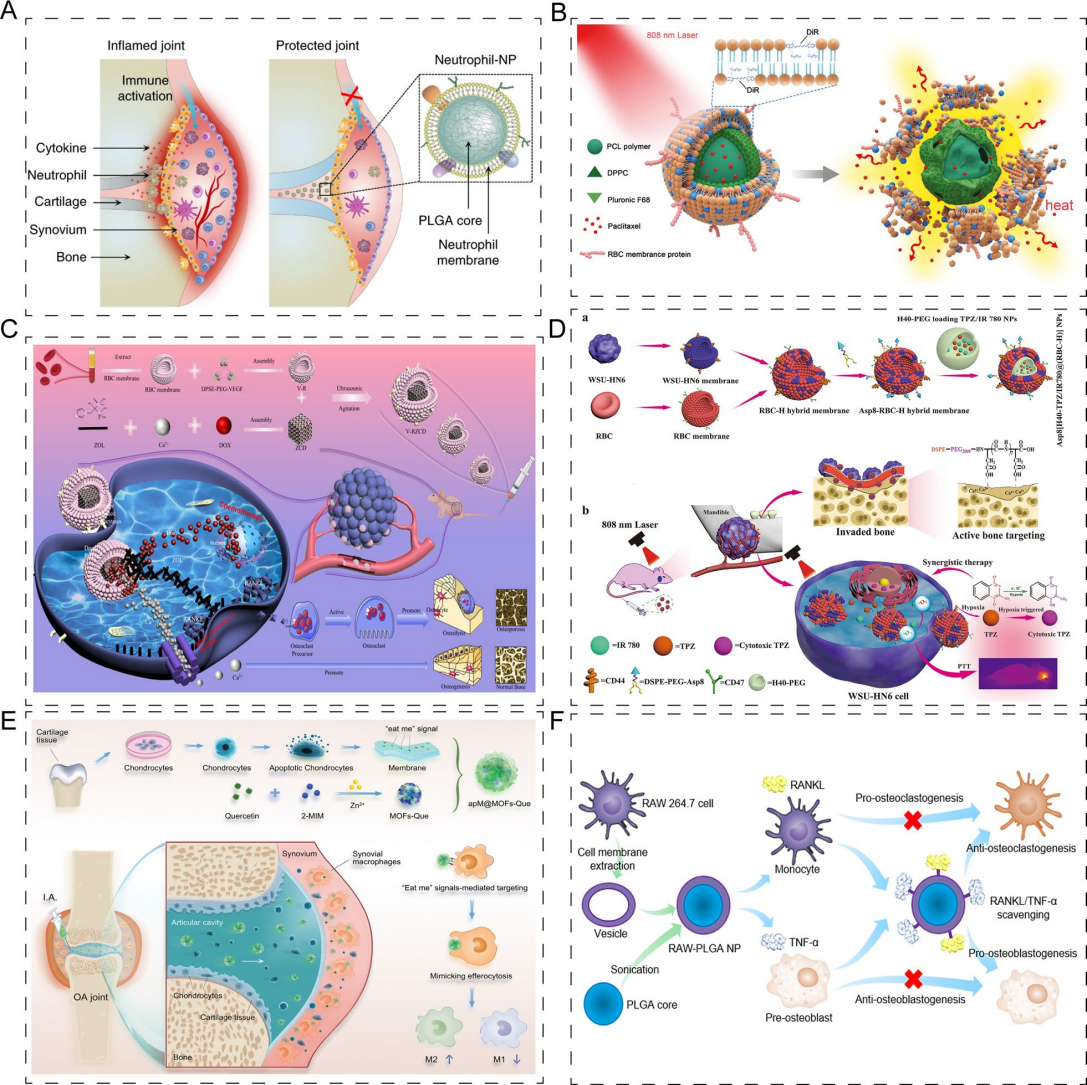
Application of membrane-coated nanoparticles in bone /cartilage regeneration. **A** Schematic illustration of neutrophil membrane-coated PLGA nanoparticles alleviated joint damage. Reprinted with permission [180]. **B** Schematic representation of infrared laser-responsive erythrocyte member NPs system. Reprinted with permission [182]. **C** The generation of erythrocyte member NPs, which also showed the ability of anti-osteolysis abilities. Reprinted with permission [183]. **D** Schematic diagram of hybrid member of neck squamous cell carcinoma WSU-HN6 cell and erythrocyte fabrication, which showed the ability to target bone. Reprinted with permission [184]. **E** The generation of apoptotic chondrocyte membrane-coated nanoparticles loaded quercetin, which capable of repolarizing macrophages from the M1 to M2, and the signals on apoptotic chondrocyte membrane made the components been phagocytized by synovial macrophages more easily. Reprinted with permission [185]. **F** The generation of RAW-PLGA nanodecoys, which could suppress osteoporosis by inhibiting osteoclastogenesis and promoting osteoblastogenesis. Reprinted with permission [187]

Cell membrane coating technology has also found application in bone and cartilage engineering. Zhang et al. [180] developed neutrophil membrane-coated PLGA nanoparticles, which act as decoys for absorbing and neutralizing neutrophil-targeted biological molecules (Fig. 13A). The authors demonstrated that these coated nanoparticles can enhance cartilage penetration and protect it by inhibiting pro-arthritogenic factors, eventually alleviating joint damage in inflammatory arthritis.

Erythrocytes, the blood cells responsible for supplying oxygen to tissues and cells, can circulate for 40–120 days in the blood vessels due to the transmembrane protein CD47 [181]. To address the release and circulation of nanoparticles, an infrared laser-responsive erythrocyte membrane nanoparticle system was developed, which controlled drug release and enhanced circulation in the blood (Fig. 13B) [182]. Wang et al. [183] modified erythrocyte membranes with the vascular endothelial growth factor receptor (VEGFR) and entrapped ZC-doxorubicin nanoparticles into the erythrocyte membrane. The camouflage provided by the erythrocyte membrane allowed the erythrocyte membrane nanoparticles to avoid being cleared efficiently. Additionally, the erythrocyte membrane nanoparticles demonstrated anti-osteolysis capability (Fig. 13C). Furthermore, a hybrid membrane composed of neck squamous cell carcinoma WSU-HN6 cells and erythrocytes showed bone-targeting ability while simultaneously loading hyperbranched polymer nanoparticles (Fig. 13D) [184].

In addition to neutrophils and erythrocytes, macrophages and chondrocytes have also been explored as potential vehicles for drug delivery. For instance, Liu et al. [185] developed quercetin-loaded nanoparticles coated with apoptotic chondrocyte membrane, which could repolarize macrophages from M1 to M2 phenotype. The signals on apoptotic chondrocyte membrane facilitated phagocytosis of nanoparticles by synovial macrophages (Fig. 13E). Similarly, different types of macrophage membrane-functionalized PCL nanofibers were found to modulate inflammation in vitro, and the M2 macrophage membrane-functionalized PCL nanofibers exhibited the strongest potential anti-inflammatory effects in vivo [186]. In addition to drug delivery, macrophage membrane-coated nanodecoys have also been utilized for cytokine/antibody clearance. For instance, nanoparticles consisting of preosteoclast membrane-coated PLGA were generated as nanodecoys for scavenging RANKL and TNF-α, which could suppress osteoporosis by inhibiting osteoclastogenesis and promoting osteoblastogenesis (Fig. 13F) [187]. Overall, the selection of an appropriate cell type for cell membrane coating is critical to achieve optimal functional properties of the coated material. Different cell types, such as stem cells, immune cells, and endothelial cells, have different functional properties that can influence the efficacy of cell membrane coating. The choice of cell type should be based on the desired functional properties of the coated material and the specific requirements of the target tissue. Future research should focus on developing innovative cell membrane coating strategies, and conducting well-designed preclinical and clinical studies to validate the safety and efficacy of cell membrane-coated materials for bone/cartilage regeneration.

#### Extracellular vesicles

**Fig. 14:**
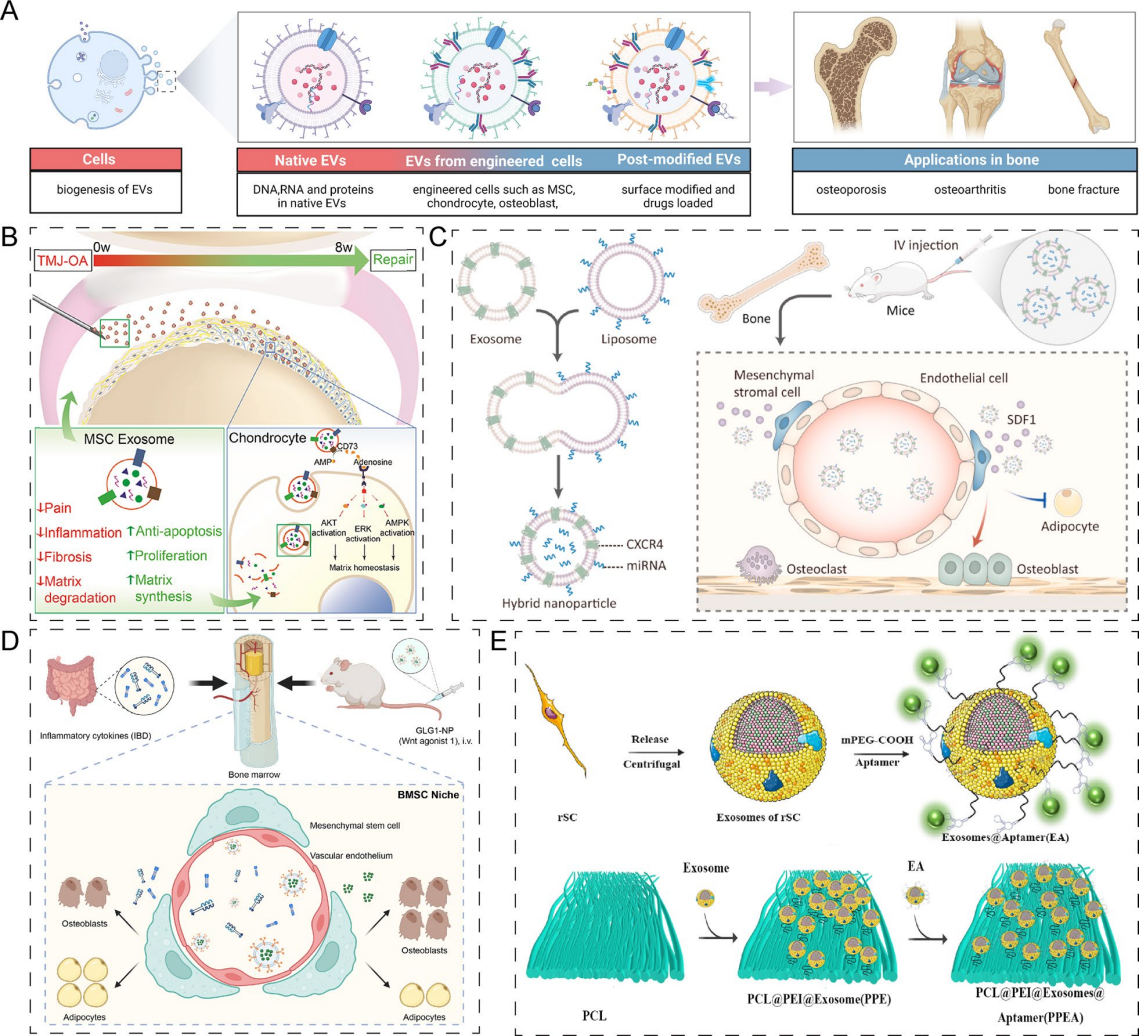
Application of native EVs, EVs from engineered cells and post modified EVs in bone/cartilage regeneration. **A** Extracellular vesicles are of cellular origin, they can be grouped into native EVs, EVs from engineered cells and post modified EVs. Their applications in bone including osteoporosis, osteoarthritis and bone fracture. Figures created with BioRender.com. **B** Native EVs from MSCs could alleviate temporomandibular joint osteoarthritis. Reprinted with permission [190]. **C** BMP2 expressed EVs secreted by HMSCs mediated HMSC osteogenic differentiation in vitro and promoted bone generation in vivo. Reprinted with permission [193]. **D** Engineered GLG1-exosomes carrying Wnt agonist 1 proved to alleviate impaired bone loss and bone fracture. Reprinted with permission [194]. **E** Aptamer engineering exosome and embedding it onto PPEA. Reprinted with permission [197]

Extracellular vesicles (EVs) are membrane-bound vesicles released by cells with a size ranging from 50 to 5000 nm. They contain proteins, nucleic acids, and lipids, which play essential roles in various biological functions [188]. Based on their size, EVs are categorized into two types, namely exosomes (50–150 nm), microvesicles (50–1000 nm) and apoptotic bodies (up to 5000 nm) (Fig. 14A). In recent years, EVs from different cell types have been extensively studied for their therapeutic potential in treating bone-related disorders such as osteoporosis, osteoarthritis (OA), and bone fractures [189]. Unmodified EVs have demonstrated promising results in OA theranostics and treatment (Fig. 14B) [190]. Mesenchymal stem cell-derived EVs (MSC-EVs) have also been extensively investigated for their therapeutic effects in preclinical studies. For example, human embryonic MSC-derived exosomes have been shown to repair and regenerate osteochondral defects when injected intra-articularly weekly [191]. Moreover, young MSC-EVs have been found to alleviate the senescence of MSCs by upregulating p21 and p16 expression [144].

To improve exosome bioactivity and endow exosomes with special functions, progenitor cells such as MSCs, chondrocytes, and osteoblasts have been engineered using pro-inflammatory factors, growth factors, transcription factors, and mechanical stimulation [192]. For example, bone morphogenetic protein 2 (BMP2)-expressed EVs secreted by HMSCs mediated HMSC osteogenic differentiation in vitro and promoted bone formation in vivo. In our previous research, we generated C-X-C motif chemokine receptor 4 (CXCR4) expressed exosomes derived from NIH-3T3 cells. CXCR4^+^ exosomes fused with liposomes which connected with antagomir-188, a microRNA promoting MSCs adipogenesis differentiation and inhibiting osteogenesis differentiation, which showed an ideal treatment for age-related bone loss (Fig. 14C) [193]. In another research, engineered GLG1(Golgi glycoprotein 1)-exosomes carrying Wnt agonist 1 proved to alleviate impaired bone loss and bone fracture caused by inflammatory bowel diseases (Fig. 14D) [194]. Generally, engineered-EVs have been used as a tool for microRNA delivery for osteoarthritis. Dai et al. [195] found that BMSCs pretreated by TGFβ3 secreted EVs were rich in miR-455 and enhanced chondrogenesis. In vivo, TGFβ3-treated EVs were embedded in hydrogel which proved to accelerate cartilage repair in situ.

Exosomes can also be functionalized directly using various approaches such as fluorescent probes, targeting peptides, and aptamers to achieve specialized applications [192]. For instance, to improve bone-targeting, Fu et al. incorporated a bone-targeting peptide onto MSC-derived exosomes. This engineered exosome carrying siShn3 demonstrated the ability to enhance osteogenic differentiation and type H vessel formation while inhibiting osteoclast formation [196]. Guo et al. engineered an aptamer-functionalized exosome that was embedded onto a PCL-PEI biomimetic periosteum (PPEA). The PPEA showed promising functions in enhancing bone regeneration and angiogenesis in vitro and in vivo by targeting injured nerves (Fig. 14E) [197].

Bacterial extracellular vesicles (BEVs) are known to transport various cargo, including cytoplasmic proteins, toxins, and nucleic acids [198]. Amont them, the gut microbiota plays a critical role in maintaining human health. Xie et al. [199] reported that cohousing with healthy mice could mitigate bone loss caused by glucocorticoid treatment. Treatment with extracellular vesicles secreted by *Lactobacillus* promoted osteogenesis, angiogenesis, and reduced cell apoptosis of animals.

However, several challenges need to be addressed to optimize the efficacy, safety, and clinical translation of EV-based therapies for bone and cartilage regeneration. One of the primary challenges is the heterogeneity of EVs, which can impact their therapeutic efficacy and specificity. Another challenge is the understanding of EV biology and the mechanisms underlying their therapeutic effects. Furthermore, the scalability and cost-effectiveness of EV-based therapies are critical for their clinical translation and commercialization. Even so, the future of EVs in bone/cartilage regeneration is promising, and ongoing research is expected to advance their therapeutic potential and enable their widespread adoption and clinical translation.

## Conclusion and future perspectives

Natural nano-based biomaterials have gained considerable attention as promising candidates for biomedical applications due to non-toxic, biocompatible, biodegradable, less expensive and abundantly available in nature. Drawing inspiration from cell units, researchers have explored the use of nucleic acids, proteins, polysaccharides, lipids, and membranes as components of bioactive materials. These materials have the potential to promote cell adhesion, proliferation, and differentiation, facilitate cell and tissue targeting, and exhibit anti-inflammatory properties, thus accelerating bone and cartilage regeneration. However, current results have not completely met clinical, and several challenges need to be addressed for their successful application in bone and cartilage regeneration.

1. Biological variability: Natural nano-based biomaterials, being derived from biological sources, exhibit inherent variability. This can affect the reproducibility of results and the predictability of their behavior in vivo.
2. lmmunogenicity: While these nano-based biomaterials are generally considered biocompatible, there is a risk of immune response, particularly when using materials derived from non-autologous sources. The immune response triggered by natural nano-based biomaterials can affect their integration and functionality within the body. Immune reactions, including inflammation and foreign body responses, may impede tissue regeneration and scaffold integration. Strategies to modulate immune responses and improve biocompatibility, such as surface modifications, immunomodulatory agents, or immunosuppressive treatments, need to be developed to promote better acceptance of the nano-based biomaterials by the host tissue.
3. Long-term stability and integration: Successful regeneration requires the long-term stability and integration of the nano-based biomaterials within the host tissue. Natural nano-based biomaterials should not only provide initial structural support but also facilitate the formation of functional tissues with appropriate extracellular matrix production, vascularization, and mechanical integrity. Achieving long-term stability and integration remains a challenge, particularly in load-bearing or dynamic tissue environments.
4. Controlled degradation: Balancing the degradation rate of these nano-based biomaterials with the rate of tissue regeneration is a significant challenge. Too fast, and the scaffold may collapse before sufficient healing; too slow, and it may impede the integration of new tissue.
5. Cost-effectiveness and accessibility: The cost-effectiveness and accessibility of natural nano-based biomaterials play a crucial role in their widespread adoption and clinical implementation. Developing efficient and cost-effective manufacturing processes, utilizing readily available raw materials, and optimizing fabrication techniques are essential for making these nano-based biomaterials more accessible to healthcare providers and patients.
6. Limitations in clinical application: The clinical application of natural biomaterials presents several limitations. These include variations in their inherent properties, potential immunogenicity and immune reactions, as well as limitations in mechanical strength and stability. Moreover, challenges related to scalability, cost-effectiveness, and regulatory approval further hinder their extensive clinical use.

In addition, the field of natural nano-based biomaterials for bone and cartilage tissue engineering is rapidly advancing, driven by innovative research and interdisciplinary collaborations. With ongoing developments in material science, surface modification techniques, additive manufacturing, and advanced characterization methods, the future of natural nano-based biomaterials holds great promise for creating functional and regenerative solutions in the field of tissue engineering.

1. 3D printing and additive manufacturing: The application of 3D printing technologies in natural nano-based biomaterials has gained significant attention. Researchers are exploring the use of natural polymers as bioinks for 3D printing, enabling the fabrication of complex structures with precise control over scaffold geometry and porosity. This approach holds great potential for patient-specific implants and regenerative medicine applications [200].
2. Bioactive coatings and surface modifications: Surface modifications of natural nano-based biomaterials are being investigated to improve cell-material interactions and promote tissue regeneration. Surface functionalization with bioactive molecules, such as growth factors or cell-binding peptides, can enhance cell adhesion, proliferation, and differentiation [201]. Additionally, surface modifications can be employed to control the release of bioactive agents from the material, providing temporal control over the regenerative process. [202].
3. Personalized medicine: With advancements in genetic engineering and synthetic biology, it may be possible to design personalized nano-based biomaterials tailored to the patient's genetic profile. For example, personalized medicine can extend to the use of patient-derived cells in combination with natural nano-based biomaterials. By utilizing induced pluripotent stem cells (iPSCs) or cells obtained through direct reprogramming, it is possible to generate patient-specific cells for tissue engineering applications. These cells can be combined with natural nano-based biomaterials to develop personalized constructs that closely mimic the patient's native tissue, promoting better integration and functional restoration.

In conclusion, while there are significant challenges to overcome, the future of natural nano-based biomaterials in tissue engineering is bright. The integration of nanotechnology and the development of novel delivery systems are expected to drive significant advancements in this field. Continued research and innovation in this area are crucial for realizing the full potential of these nano-based biomaterials in bone and cartilage regeneration.

## Data Availability

Not applicable.

## Abbreviations

HA: Hydroxyapatite
VEGF: Vascular endothelial growth factor
DNA: Deoxyribonucleic acid
RNA: Ribonucleic acid
Col10a1: Collagen type X alpha 1 chain
SELEX: Systematic evolution of ligands by exponential amplification
PEG: Polyethylene glycol
MSCs: Mesenchymal stem cells
PCL: Poly-ε-caprolactone
tFNAs: Tetrahedral framework nucleic acids
FGF2: Fibroblast growth factor 2
ECM: Extracellular matrix
APC: Mineralization, stabilizing amorphous calcium
COL: Collagen
SF: Silk fibroin
TGF-β1: Transforming growth factor-β1
BMPs: Bone morphogenetic proteins
BFP-1: Bone-forming peptide-1
CAP: Chondrocyte-affinity peptide
RGD: Arg-Gly-Asp
ADSCs: Adipose-derived stem cells
OA: Osteoarthritis
qPCR: Quantitative polymerase chain reaction
CS: Chondroitin sulfate
AG: Alginate/gellan gum
VEGFR: Vascular endothelial growth factor receptor
HE: Hematoxylin and eosin staining
COL2: Type II collagen
BMP2: Bone morphogenetic protein-2
SSCs: Skeletal stem cells
Micro-CT: Micro-computed tomography
rBMSCs: Rat bone-marrow-derived MSCs
p-OGP: Phosphorylated osteopontin
